# Supplementary material for: General synthesis of ionic-electronic coupled two-dimensional materials
Source: Nat Commun. 2024 May 22;15:4368. doi: 10.1038/s41467-024-48690-7 (PMC11111738; doi:10.1038/s41467-024-48690-7)
Supplement: Supplementary file 1 — Supplementary Information [file 41467_2024_48690_MOESM1_ESM.pdf]

Supplementary Information

General synthesis of ionic-electronic coupled two-dimensional materials

Xiang Xu[1, #], Yunxin Chen[1, #], Pengbin Liu[1], Hao Luo[2], Zexin Li[1], Dongyan Li[1], Haoyun Wang[1], Xingyu Song[1], Jinsong Wu[2], Xing Zhou[1✉] and Tianyou Zhai[1, 3✉]

[1]State Key Laboratory of Materials Processing and Die & Mould Technology, School of Materials Sciences and Engineering, Huazhong University of Science and Technology, Wuhan 430074, P. R. China

[2]Nanostructure Research Center, State Key Laboratory of Advanced Technology for Materials Synthesis and Processing, Wuhan University of Technology, Wuhan 430070, P. R. China.

[3]Optics Valley Laboratory, Hubei 430074, P. R. China

[#]These authors contributed equally.

[✉]Email: zhoux0903@hust.edu.cn (X.Z.); zhaity@hust.edu.cn (T.Y.Z.)

Supplementary Figures

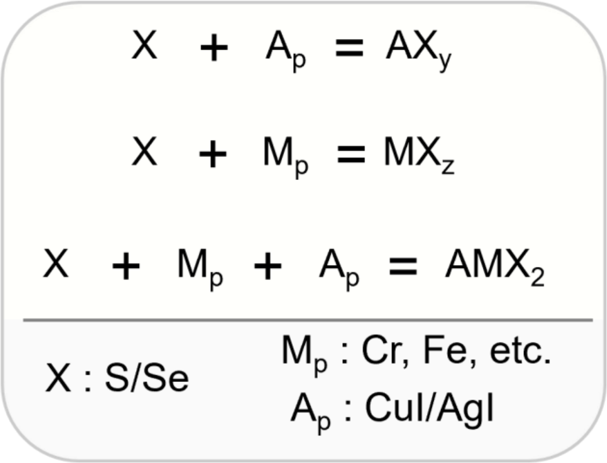

Supplementary Figure 1. Chemical reactions that may occur during the synthesis process of AMX_2.

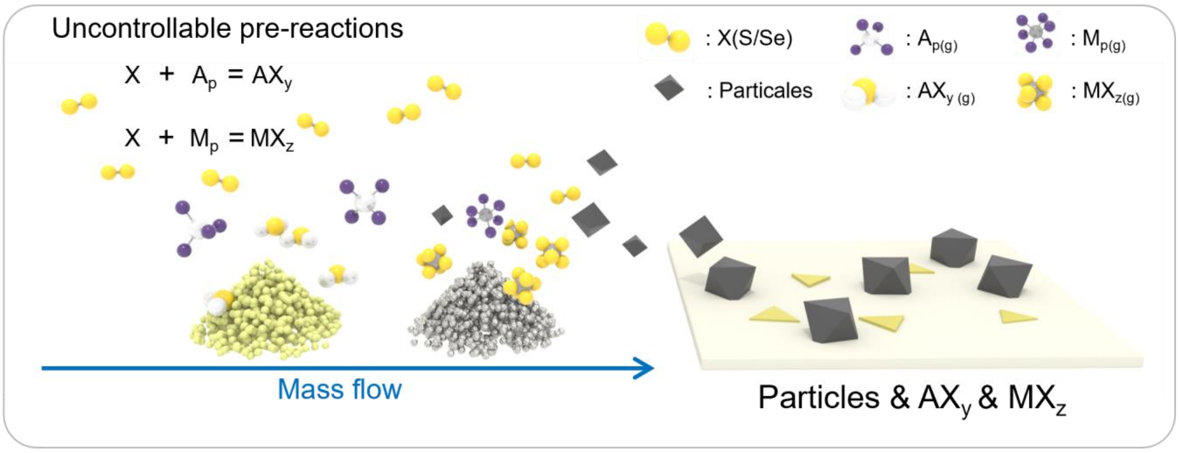

Supplementary Figure 2. Uncontrollable pre-reaction in the common CVD method for synthesizing AMX_2.

In the common chemical vapor deposition (CVD) process, the vapor of chalcogen precursor and metal precursors are both placed upstream. The precursors have already met before reaching the substrate and undergo uncontrollable pre-reactions during the heating and mass transport process. Due to the uncontrollable pre-reactions, large numbers of binary byproducts AX_y and MX_z are generated, as well as particulate matter generated through heterogeneous reactions during mass transportation. These complex processes result in the nonuniform distribution of precursors and uncontrollable synthesis of 2D AMX_2.

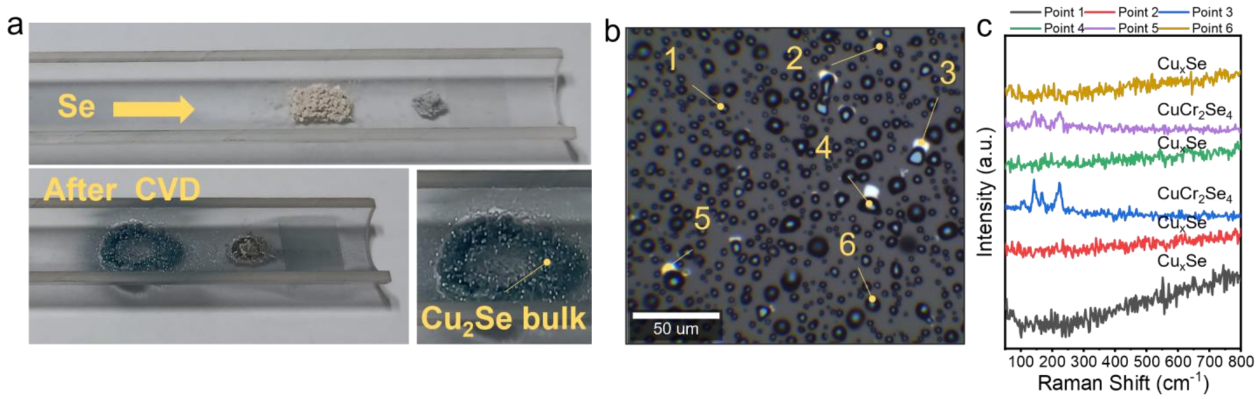

Supplementary Figure 3. Synthesis of $CuCrSe_2$ by common CVD method. a Change of precursors before and after the reaction. **b** Typical optical image of the growth situation using this method. **c** Raman spectra of each point in (**b**).

CuI and Cr powders are chosen as two types of metal precursors. The mica substrate is placed downstream, and Se vapor comes from upstream. During this CVD process, CuI and Cr powders are excessively selenated, hindering the further evaporation of the precursor. Large numbers of bulks like Cu_xSe by-products and a small amount of other stoichiometric ternary by-products such as $CuCr_2Se_4$ are generated on the substrate.

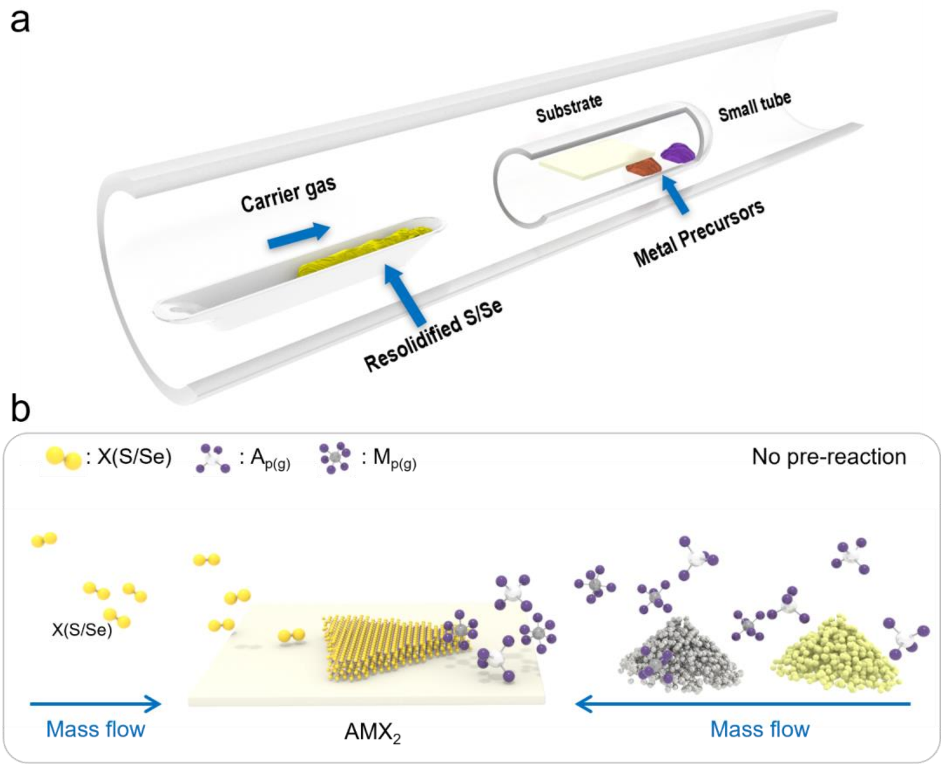

Supplementary Figure 4. Schematic diagrams of separated-precursor-supply CVD method. a CVD setup for synthesizing AMX_2 nanosheets. **b** Schematic diagram of the separated precursor transport mode.

Resolidified chalcogen precursors were obtained by annealing sulfur/selenium powder in Ar at 150℃/300℃ for 5 min in a tube furnace and then cooling to room temperature naturally.

In the separated-precursor-supply CVD method, the metal precursors were placed in a small quartz tube with one side sealed. This CVD setup protects the metal precursors and forces the vaporized metal precursors to flow against the S/Se vapors, which suppress the pre-reactions between the precursors. Consequently, the by-products also can be suppressed and the supply of metal precursors could maintain a stable state during the whole process to support the synthesis of the AMX_2 nanosheets.

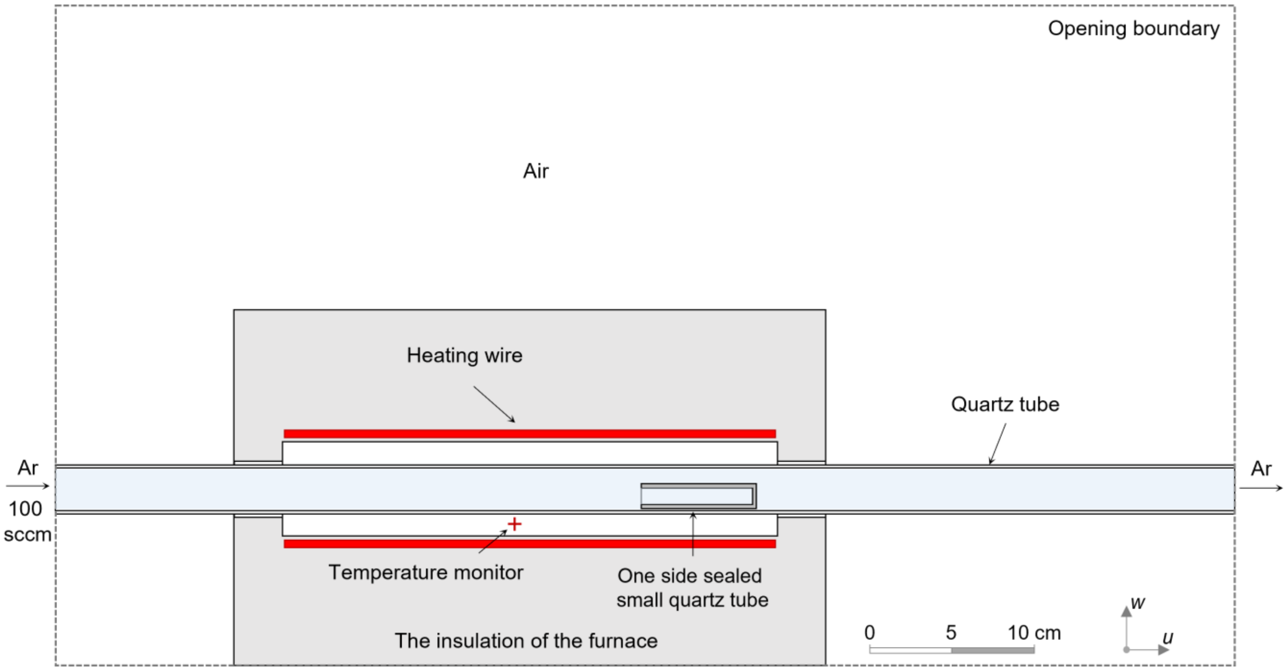

Supplementary Figure 5. Experiment setup of CVD tube furnace for computational fluid dynamics simulations.

In this CFD simulation, there are two fluid domains, including the space in the quartz tube and the space out of the quartz tube. The size of the whole model is 72*50*50 cm, and the boundary walls (dashed gray lines) are set as openings with fixed pressure and temperature. The total number of elements and nodes of the modeling are 1821120 and 428231, respectively. In the quartz tube, the Ar is injected through the inlet hole (radius: 2 mm) with a constant flow rate of 0.13 m/s, which is the same as the experiment Ar flux of 100 sccm. The temperature monitor point is at the real position of the thermocouple. The two solid domains are the quartz tube and the insulation of the furnace. We defined the parameters for the quartz and the insulation of the furnace which are given in the following table.

	**Density**	**Heat capacity**	**Thermal conductivity**
Quartz	2210 kg/m^3	892 J/Kg K	1.7 W/m K
The insulation of the furnace	300 kg/m^3	120 J/Kg K	0.14 W/m K

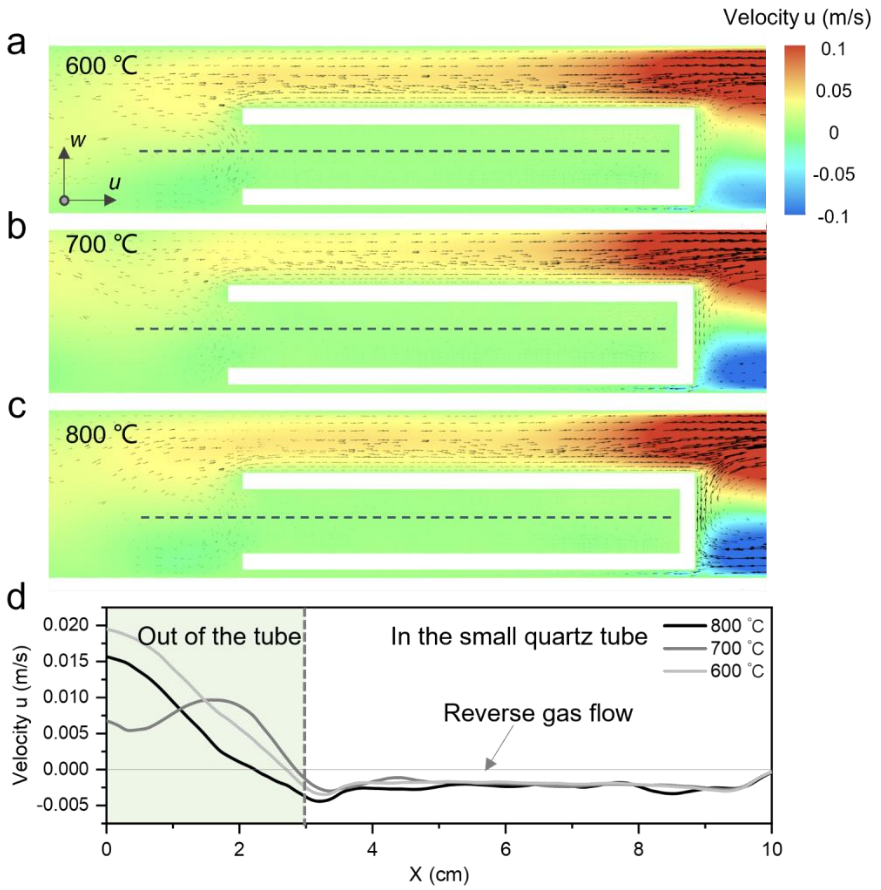

Supplementary Figure 6. The velocity distribution around the small quartz tube at different temperatures. a-c Distribution of velocity u in the small quartz tube at 600℃, 700℃, and 800℃ respectively. **b** The variation curve of velocity u along the dashed line in (**a**)-(**c**).

The flow rate in the outer space of the tube is relatively high, and the *u* component is positive. Due to the one-sided sealing of the small quartz tube, the flow rate in the tube rapidly decreases, indicating that the gas flow is very stable. The velocity component in the *u* direction is mainly negative, showing that the gas flow inside the tube is mainly reversed. This velocity distribution ensures the stable supply of the metal precursors. And this distribution has no obvious changes at 600℃-700℃, which means that this method allows us to realize a stable supply of metal precursors when synthesizing the 20 AMX_2.

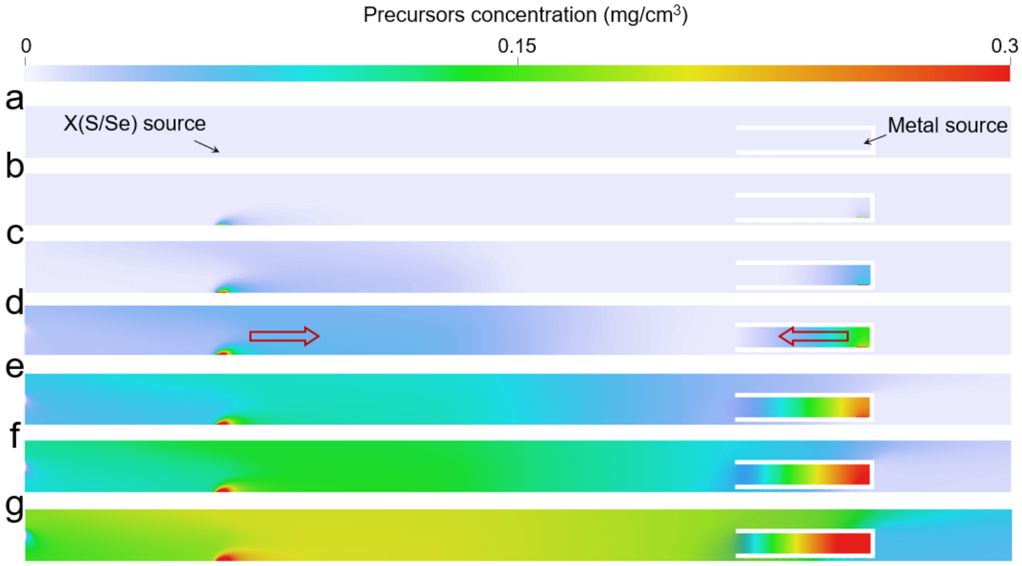

Supplementary Figure 7. CFD transient simulation of precursor concentration. a-g Precursor distribution at time steps 1, 5, 9, 12, 17, 22, and 37 of transient simulation, respectively.

The mass flow direction inside the small quartz tube is opposite to that of X(S/Se) vapor according to the changing of precursor concentration over time. In addition, the metal concentration inside the small tube can reach a high level to ensure a matched concentration between metal and chalcogen vapors.

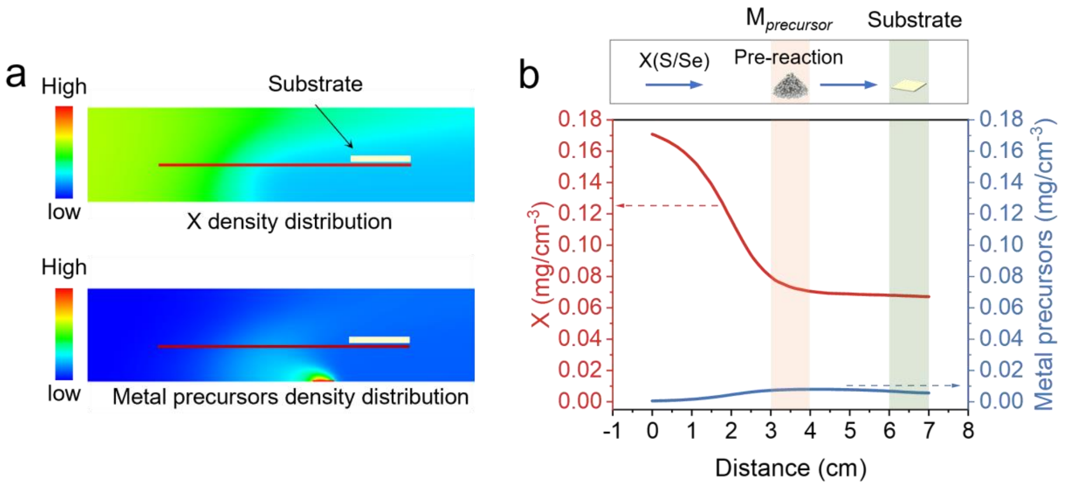

Supplementary Figure 8. Distribution of precursor concentration in the common CVD method. **a** precursor concentration distribution near the substrate. **b** Precursor concentration along the red solid line in (**a**).

The vapor of X(S/Se) meets with the metal precursors before reaching the substrate, leading to uncontrollable pre-reactions. Meanwhile, the concentration of the X(S/Se) precursor is much higher than that of the metal precursor, resulting in excessive selenization or sulfurization of the metal precursor.

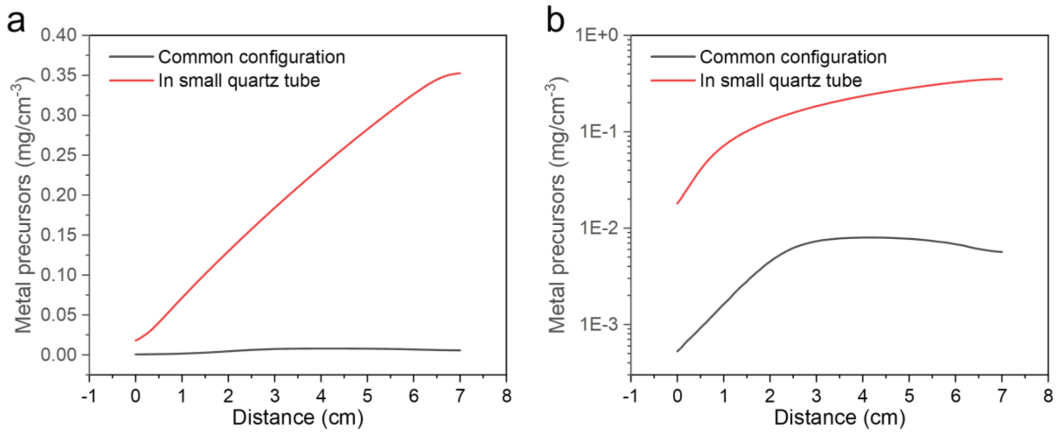

Supplementary Figure 9. Comparison of metal precursor concentration distribution of separated-precursor-supply strategy and common CVD method. The concentration of metal precursors along the red line in Fig. 1e and Supplementary Fig. 8. (**a** Linear y-axis. **b** Logarithmic y-axis.)

The concentration of metal precursors placed in small tubes is higher and the concentration distribution changes linearly, which is more conducive to achieving a sufficient and controllable supply of metal precursors.

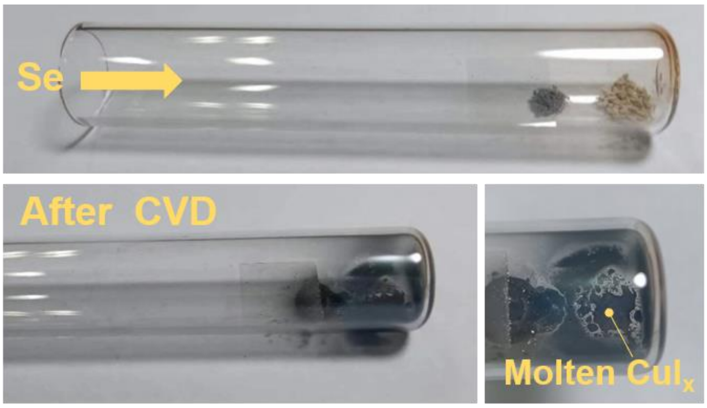

Supplementary Figure 10. Synthesis of $CuCrSe_2$ by separated-precursor-supply CVD method. Change of precursors before and after the reaction. The uniform phase and morphology distribution of the flakes can be seen in Figure 1d and 1e.

Consistent with the common CVD method, CuI and Cr powders are used as two types of metal precursor powders. The metal precursor powders are placed at the bottom of a small quartz tube with one side sealed, and the fluorocrystalline mica substrate is placed near the metal precursors. After the CVD process, CuI powder was not excessively selenized and remained in a molten and resolidified state. Importantly, we obtained uniform $CuCrSe_2$ nanosheets on the mica substrate (Figure 1d and 1e).

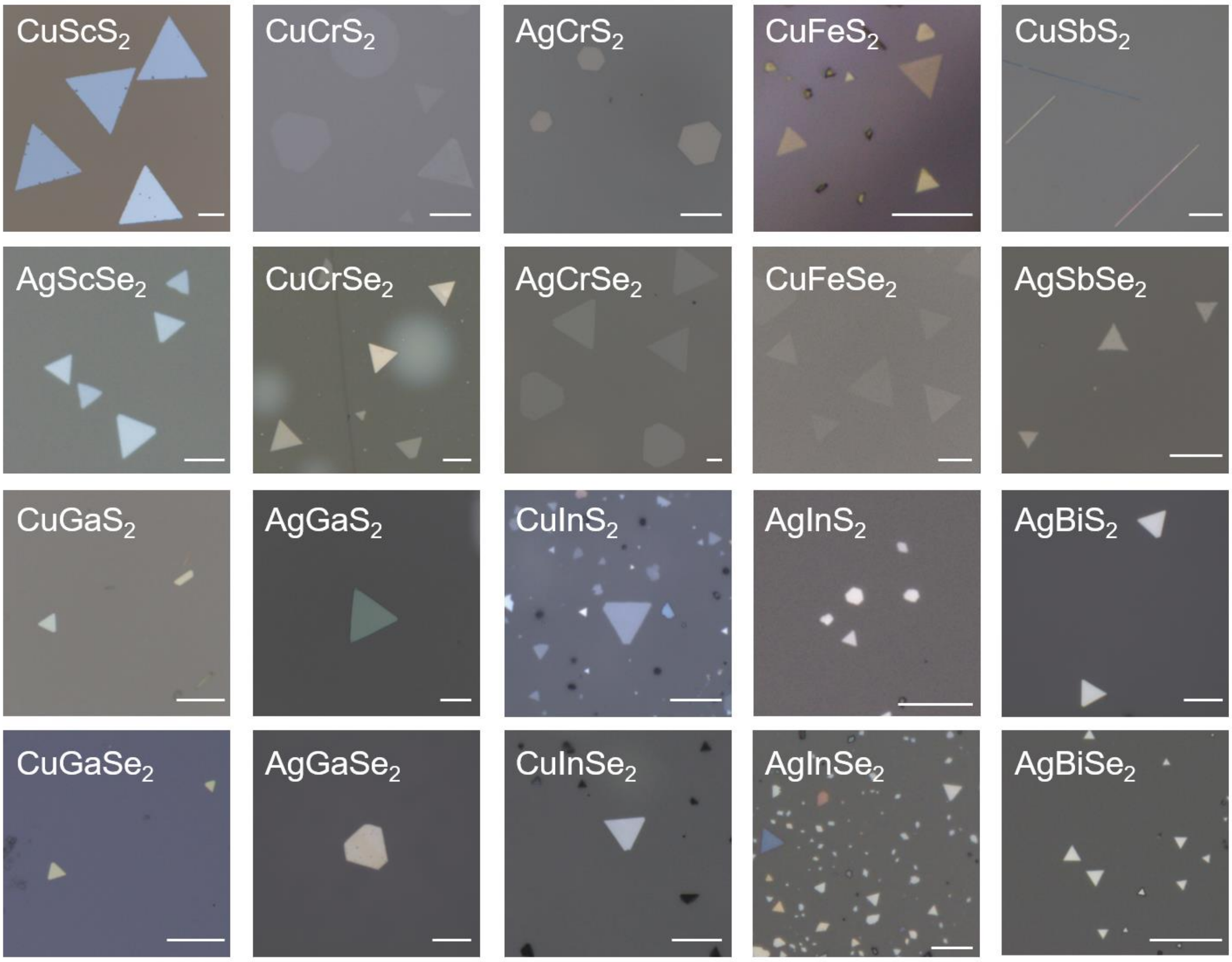

Supplementary Figure 11. Typical optical images of the 20 kinds of 2D AMX_2 on the substrate, scale bar: 10 μm.

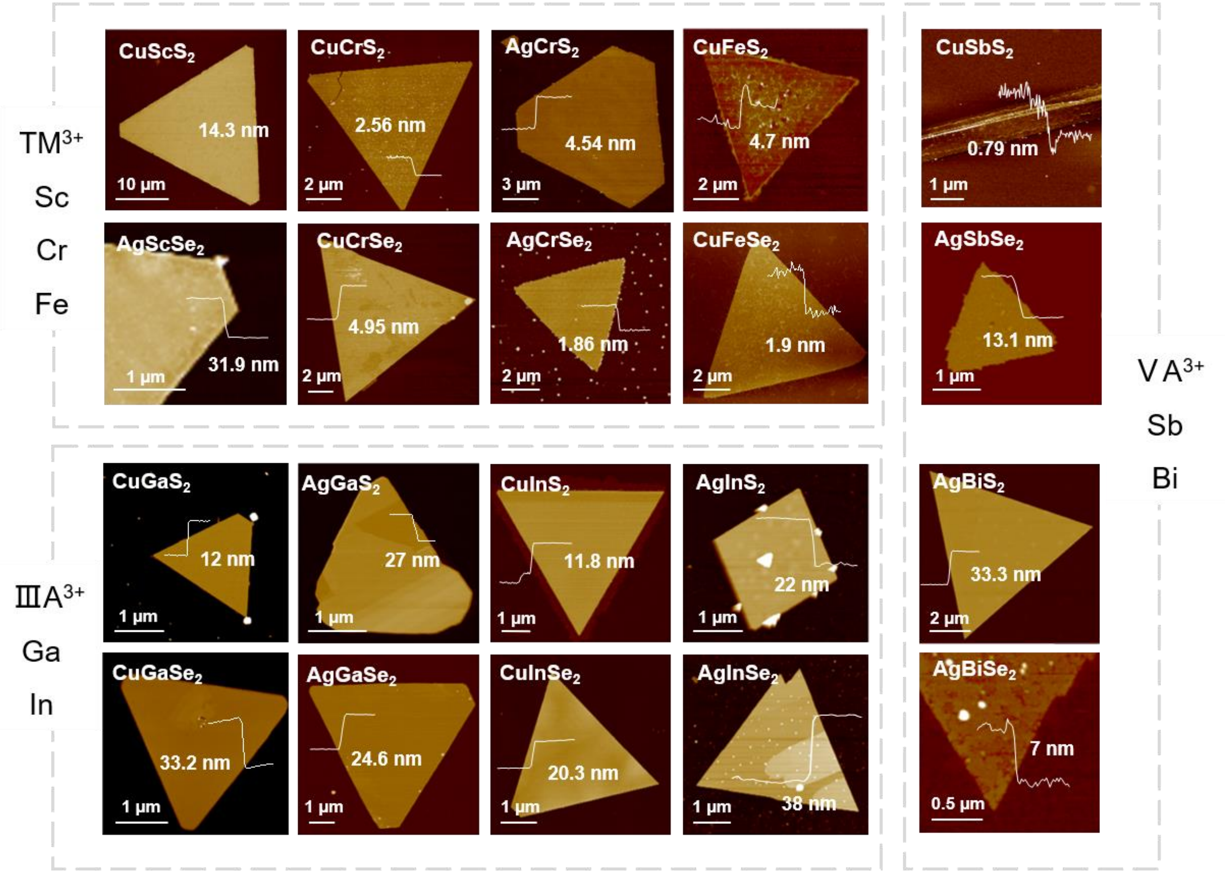

Supplementary Figure 12. AFM images of the 20 kinds of 2D AMX_2.

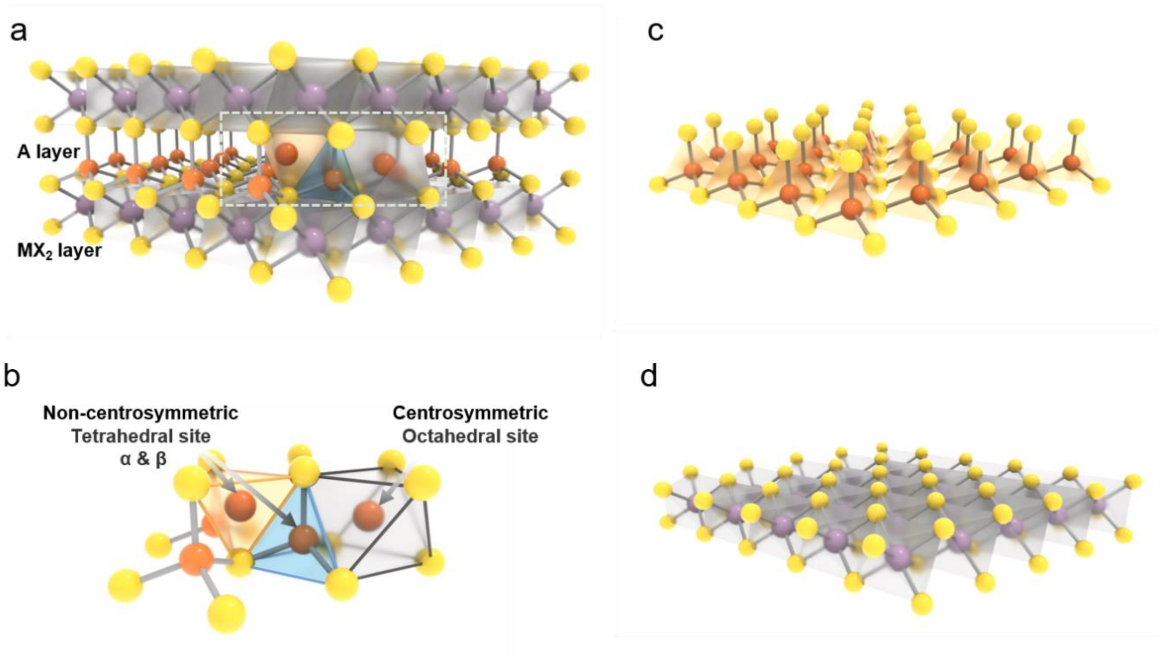

Supplementary Figure 13. The quasi-2D layered structure characteristics of AMX_2. a A atoms are limited between the MX_2 layers. **b** Tetrahedral and octahedral site of A atoms in AMX_2. **c** The tetrahedral $[AX_4]$ layer. **d** The octahedral $[MX_6]$ layer.

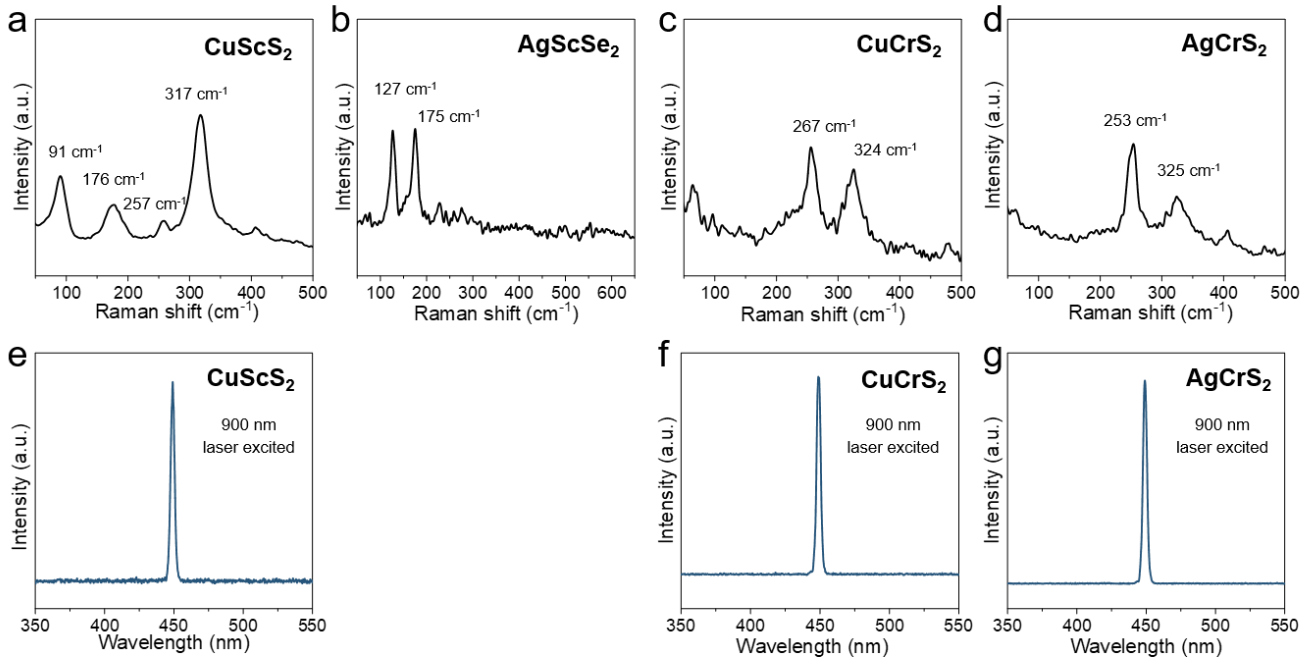

Supplementary Figure 14. Raman and SHG characterizations of $CuScS_2$, $AgScSe_2$, $CuCrS_2$, and $AgCrS_2$. a-d Raman spectra of as-synthesized $CuScS_2$, $AgScSe_2$, $CuCrS_2$ and $AgCrS_2$, respectively. **e-g** The SHG signal of $CuScS_2$, $CuCrS_2$ and $AgCrS_2$, respectively. The SHG signal was excited by a 900 nm femtosecond laser.

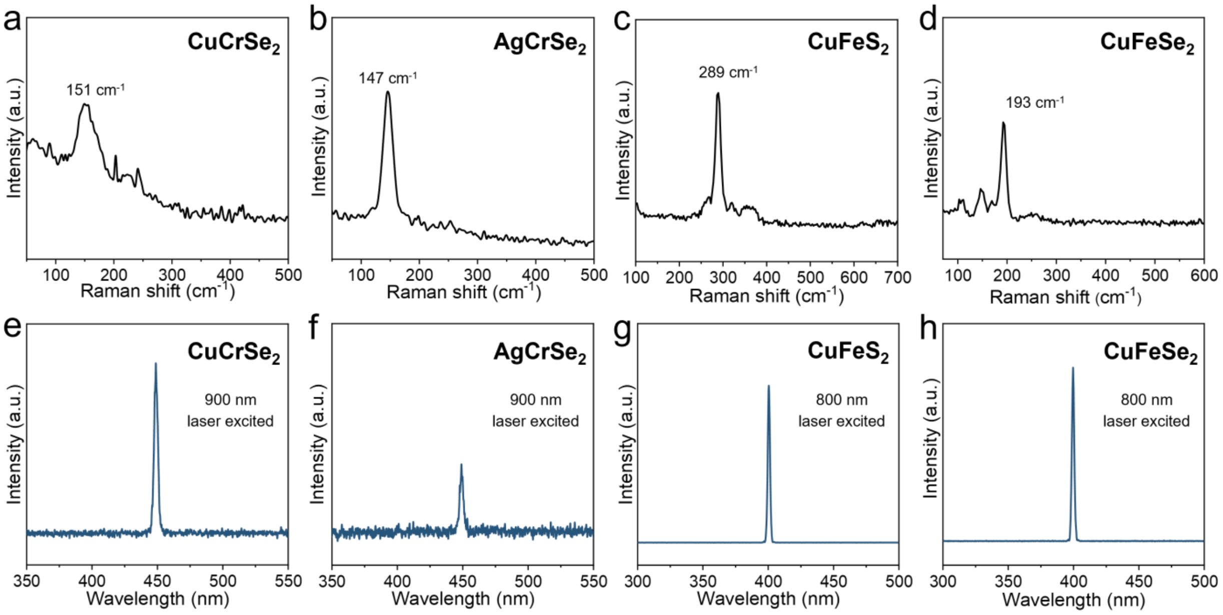

Supplementary Figure 15. Raman and SHG characterizations of $CuCrSe_2$, $AgCrSe_2$, $CuFeS_2$, and $CuFeSe_2$. a-d Raman spectra of as-synthesized $CuCrSe_2$, $AgCrSe_2$, $CuFeS_2$ and $CuFeSe_2$, respectively. **e-h** The SHG signal of $CuCrSe_2$, $AgCrSe_2$, $CuFeS_2$ and $CuFeSe_2$, respectively. The SHG signal was excited by a 900/800 nm femtosecond laser.

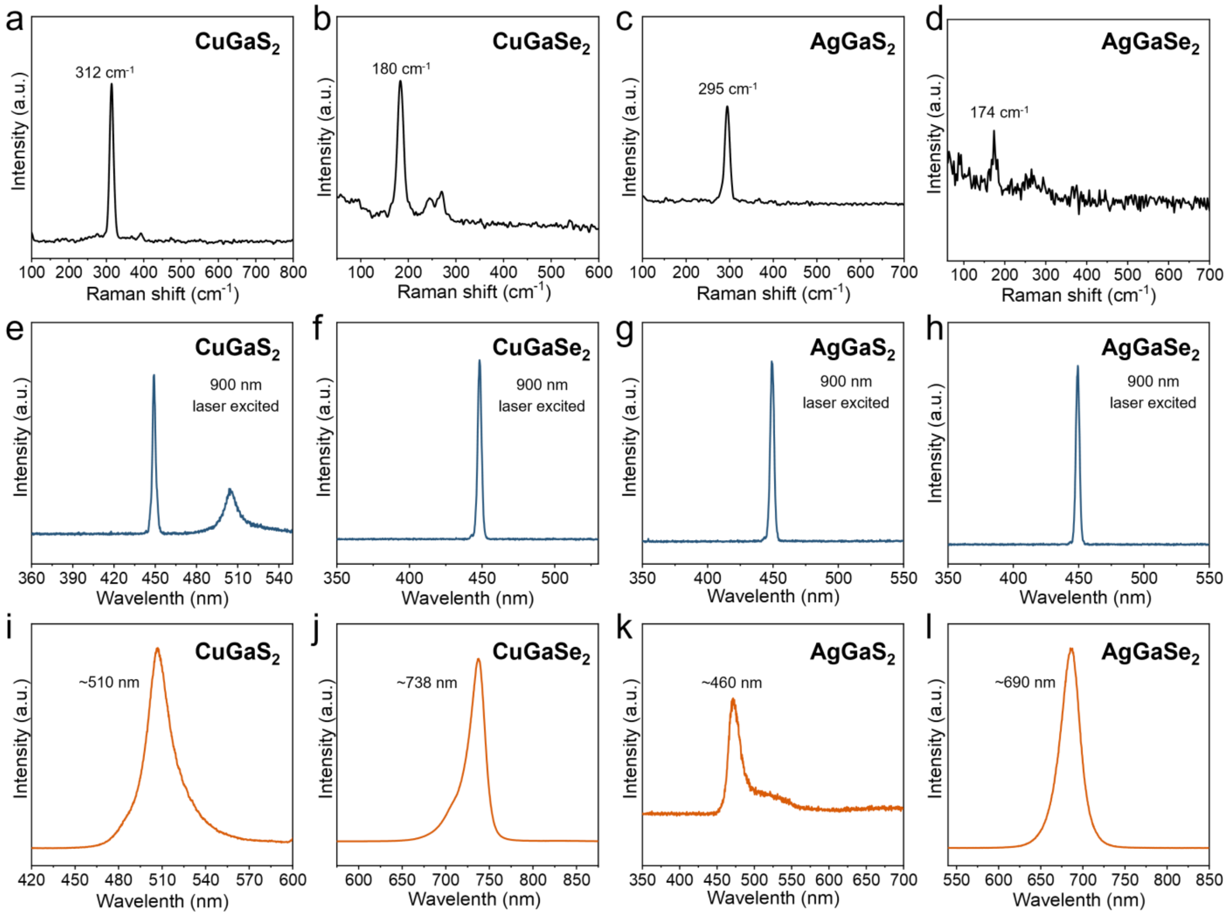

Supplementary Figure 16. Raman, SHG, and PL characterizations of $CuGaS_2$, $CuGaSe_2$, $AgGaS_2$, and $AgGaSe_2$. a-d Raman spectra of as-synthesized $CuGaS_2$, $CuGaSe_2$, $AgGaS_2$, and $AgGaSe_2$, respectively. **e-h** The SHG signal of $CuGaS_2$, $CuGaSe_2$, $AgGaS_2$, and $AgGaSe_2$, respectively. The SHG signal was excited by a 900 nm femtosecond laser. **i-l** The PL spectra of as-synthesized $CuGaS_2$, $CuGaSe_2$, $AgGaS_2$, and $AgGaSe_2$, respectively.

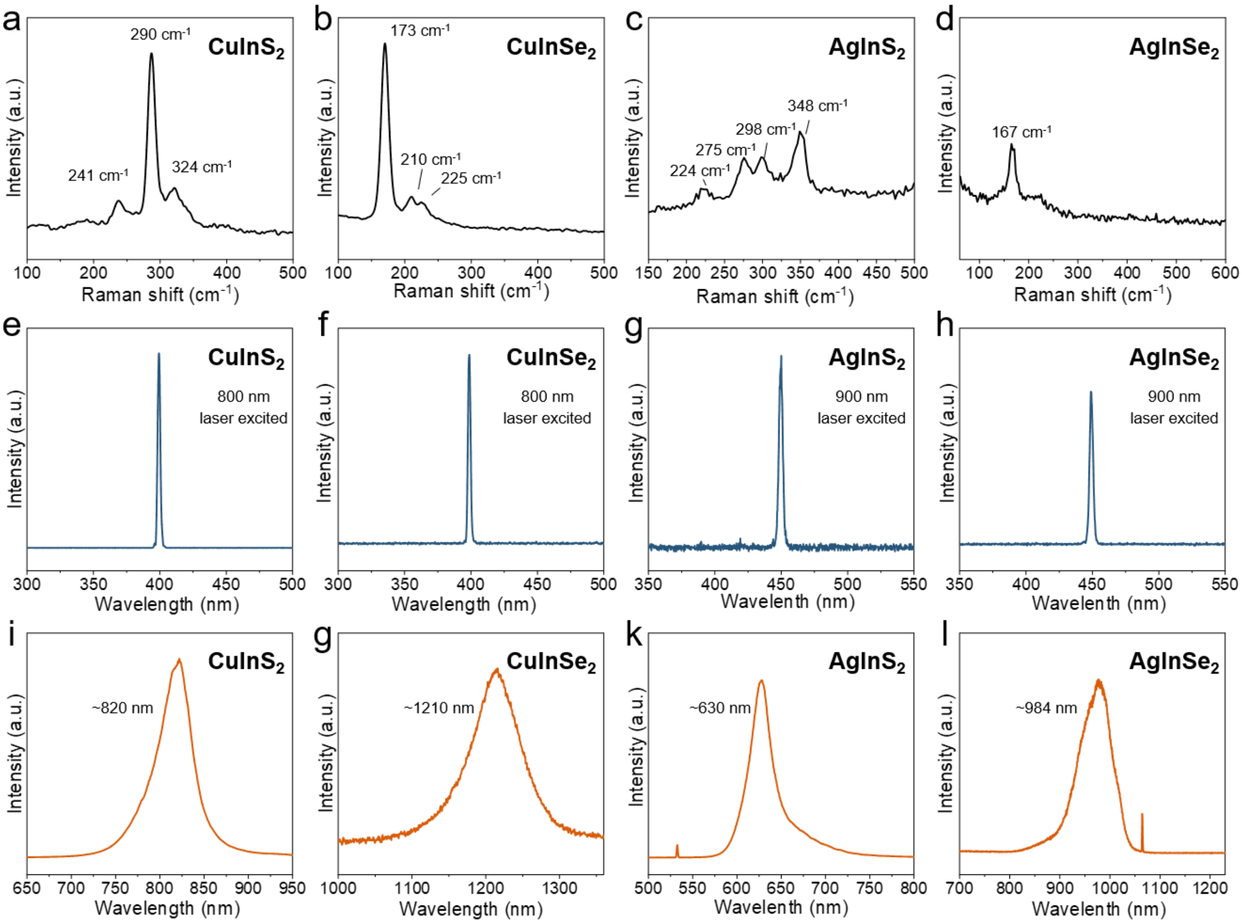

Supplementary Figure 17. Raman, SHG, and PL characterizations of $CuInS_2$, $CuInSe_2$, $AgInS_2$, and $AgInSe_2$. a-d Raman spectra of as-synthesized $CuInS_2$, $CuInSe_2$, $AgInS_2$, and $AgInSe_2$, respectively. **e-h** The SHG signal of $CuInS_2$, $CuInSe_2$, $AgInS_2$, and $AgInSe_2$, respectively. The SHG signal was excited by a 900/800 nm femtosecond laser. **i-l** The PL spectra of as-synthesized $CuInS_2$, $CuInSe_2$, $AgInS_2$, and $AgInSe_2$, respectively.

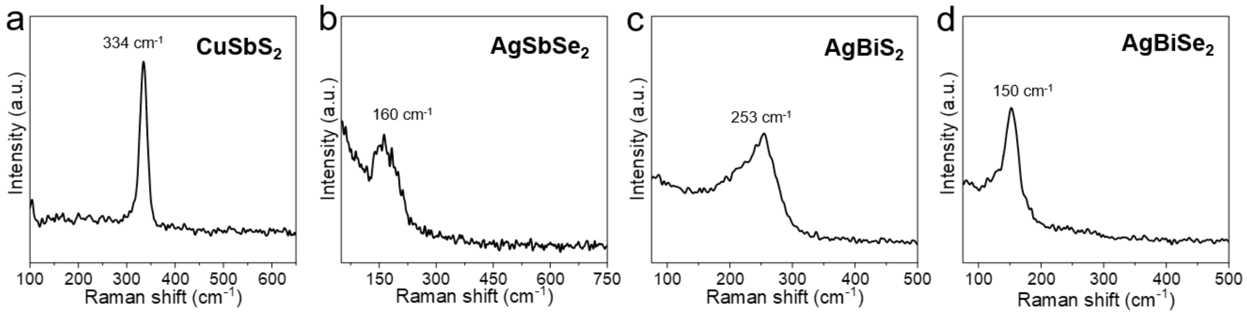

Supplementary Figure 18. Raman characterizations of $CuSbS_2$, $AgSbSe_2$, $AgBiS_2$, and $AgBiSe_2$. **a-d** Raman spectra of as-synthesized $CuSbS_2$, $AgSbSe_2$, $AgBiS_2$, and $AgBiSe_2$, respectively.

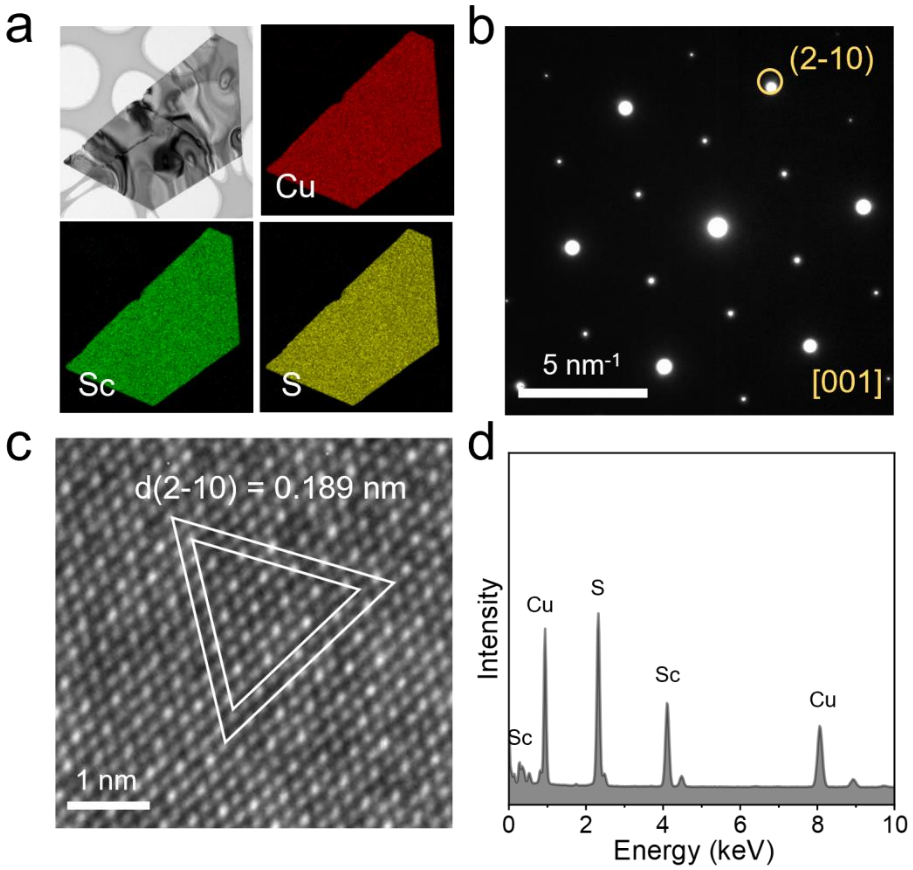

Supplementary Figure 19. Structure characterization of $CuScS_2$. a Low magnification TEM image and corresponding EDS elemental mapping of $CuScS_2$. **b** SAED pattern of as-synthesized 2D $CuScS_2$ nanosheet. **c** Atom-resolution experimental TEM image of $CuScS_2$. **d** The EDS spectra of the flake.

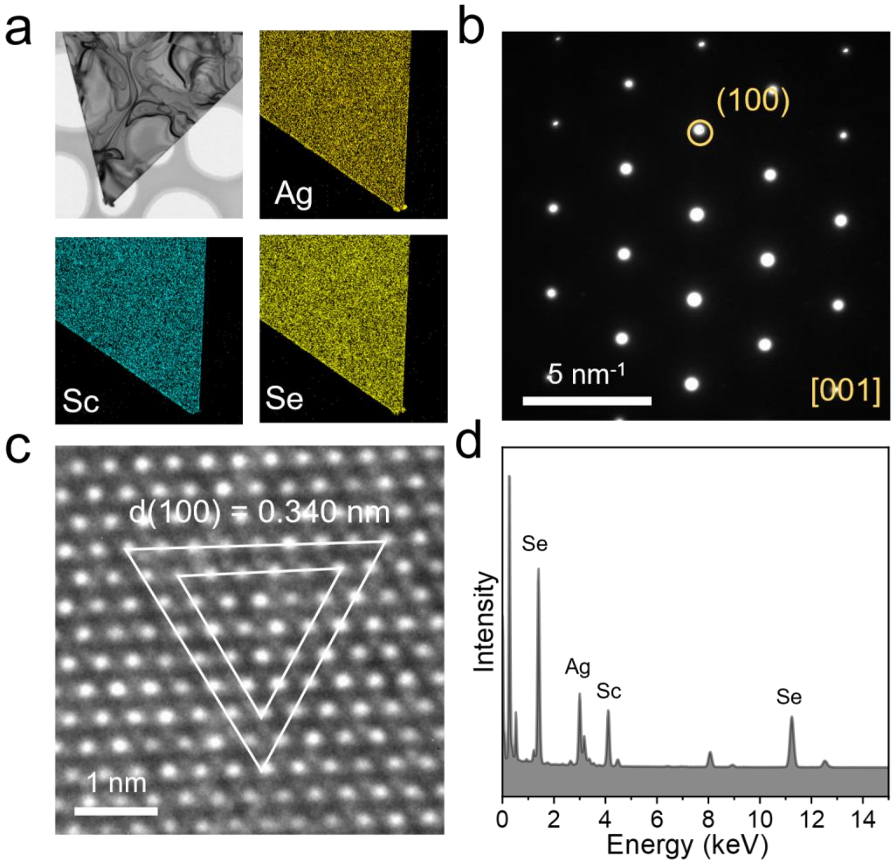

Supplementary Figure 20. Structure characterization of $AgScSe_2$. **a** Low magnification TEM image and corresponding EDS elemental mapping of $AgScSe_2$. **b** SAED pattern of as-synthesized 2D $AgScSe_2$ nanosheet. **c** Atom-resolution experimental TEM image of $AgScSe_2$. **d** The EDS spectra of the flake.

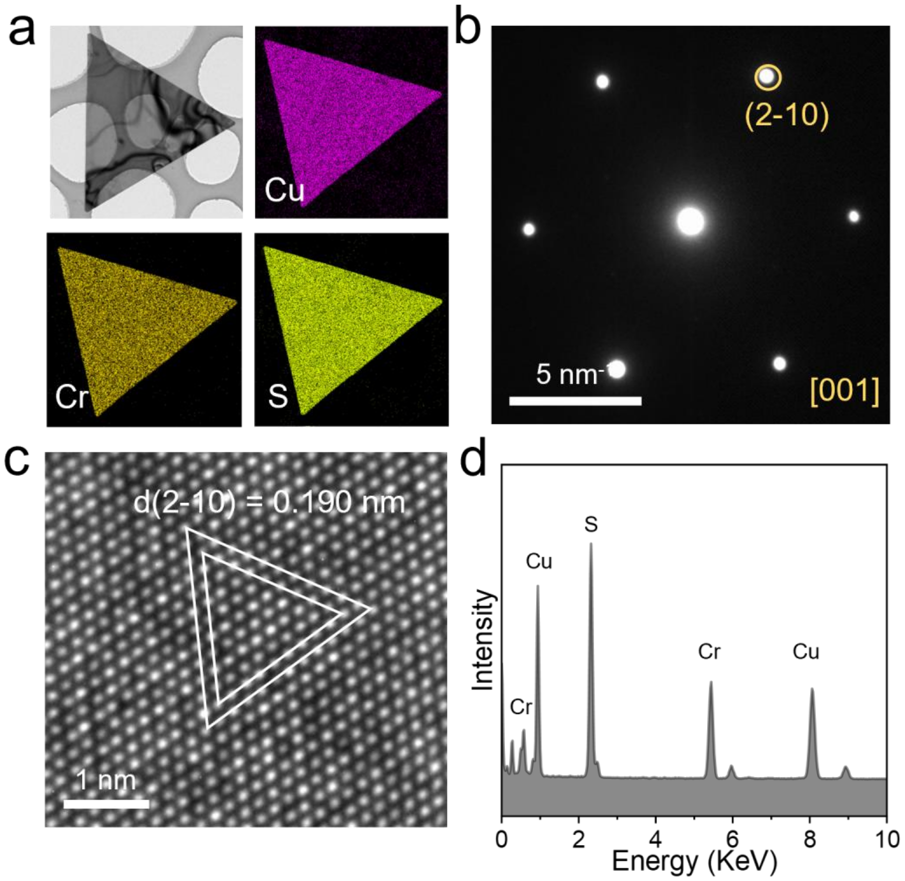

Supplementary Figure 21. Structure characterization of $CuCrS_2$. a Low magnification TEM image and corresponding EDS elemental mapping of $CuCrS_2$. **b** SAED pattern of as-synthesized 2D $CuCrS_2$ nanosheet. **c** Atom-resolution experimental TEM image of $CuCrS_2$. **d** The EDS spectra of the flake.

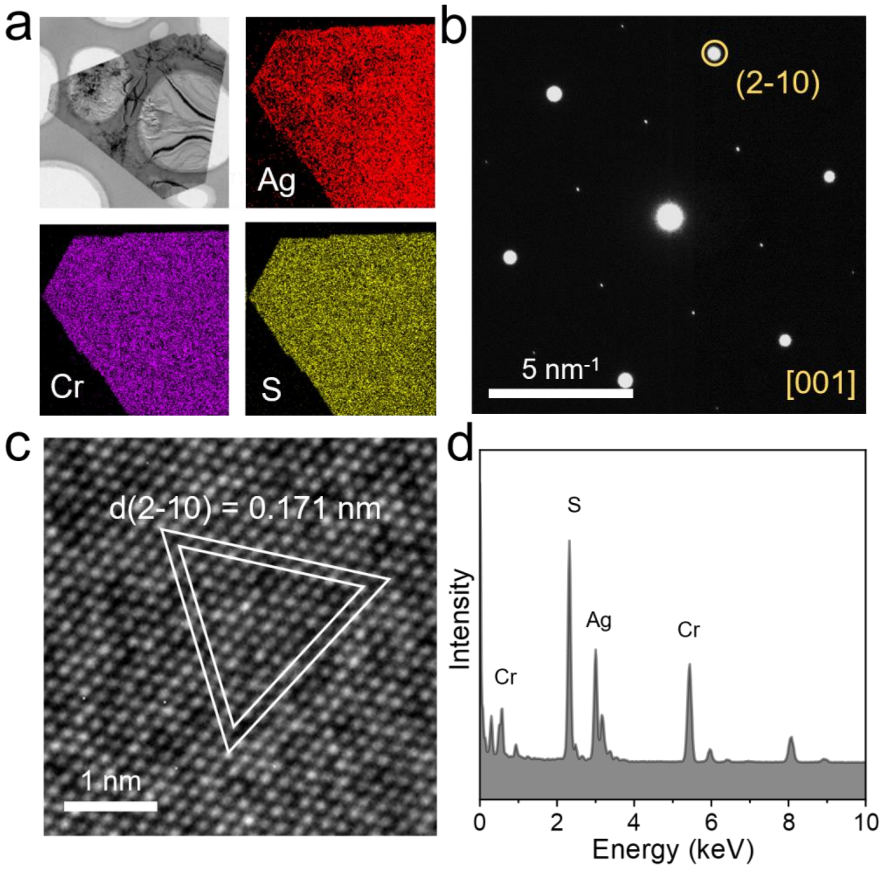

Supplementary Figure 22. Structure characterization of $AgCrS_2$. a Low magnification TEM image and corresponding EDS elemental mapping of $AgCrS_2$. **b** SAED pattern of as-synthesized 2D $AgCrS_2$ nanosheet. **c** Atom-resolution experimental TEM image of $AgCrS_2$. **d** The EDS spectra of the flake.

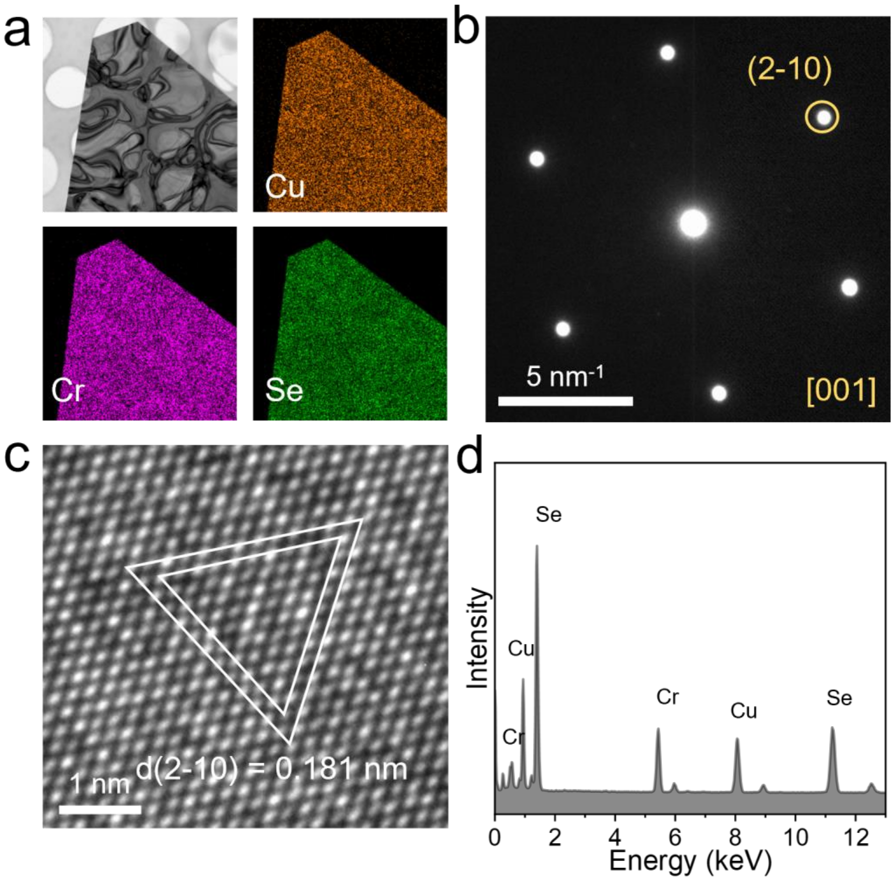

Supplementary Figure 23. Structure characterization of $CuCrSe_2$. a Low magnification TEM image and corresponding EDS elemental mapping of $CuCrSe_2$. **b** SAED pattern of as-synthesized 2D $CuCrSe_2$ nanosheet. **c** Atom-resolution experimental TEM image of $CuCrSe_2$. **d** The EDS spectra of the flake.

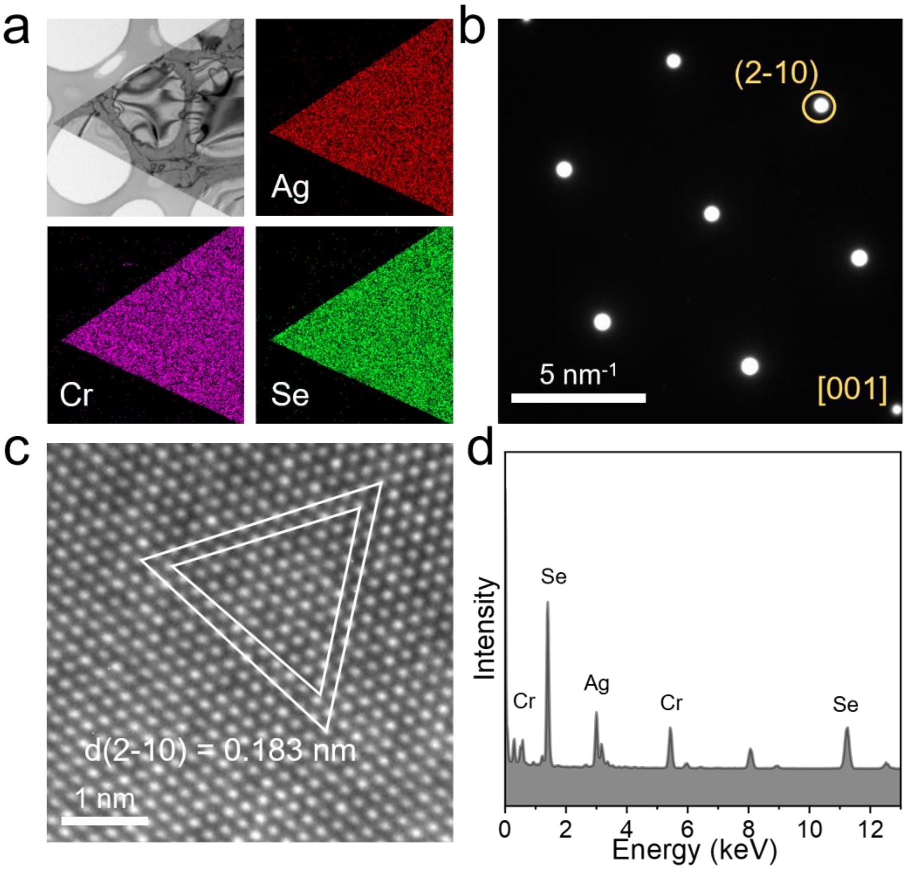

Supplementary Figure 24. Structure characterization of $AgCrSe_2$. a Low magnification TEM image and corresponding EDS elemental mapping of $AgCrSe_2$. **b** SAED pattern of as-synthesized 2D $AgCrSe_2$ nanosheet. **c** Atom-resolution experimental TEM image of $AgCrSe_2$. **d** The EDS spectra of the flake.

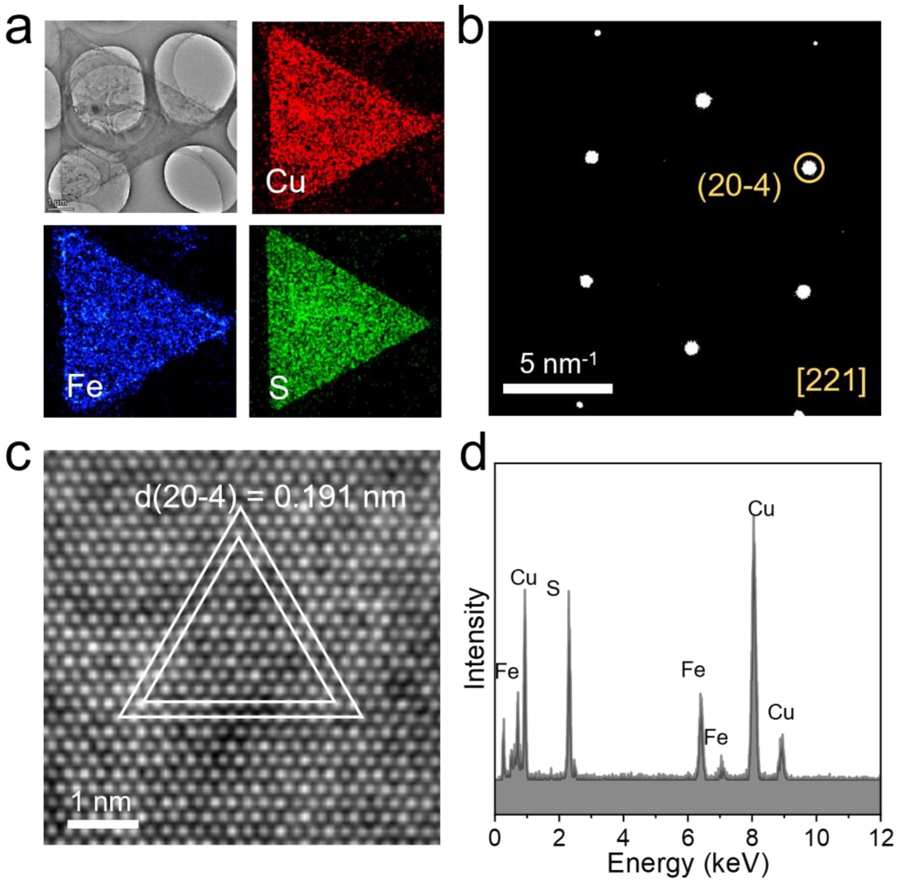

Supplementary Figure 25. Structure characterization of $CuFeS_2$. a Low magnification TEM image and corresponding EDS elemental mapping of $CuFeS_2$. **b** SAED pattern of as-synthesized 2D $CuFeS_2$ nanosheet. **c** Atom-resolution experimental TEM image of $CuFeS_2$. **d** The EDS spectra of the flake.

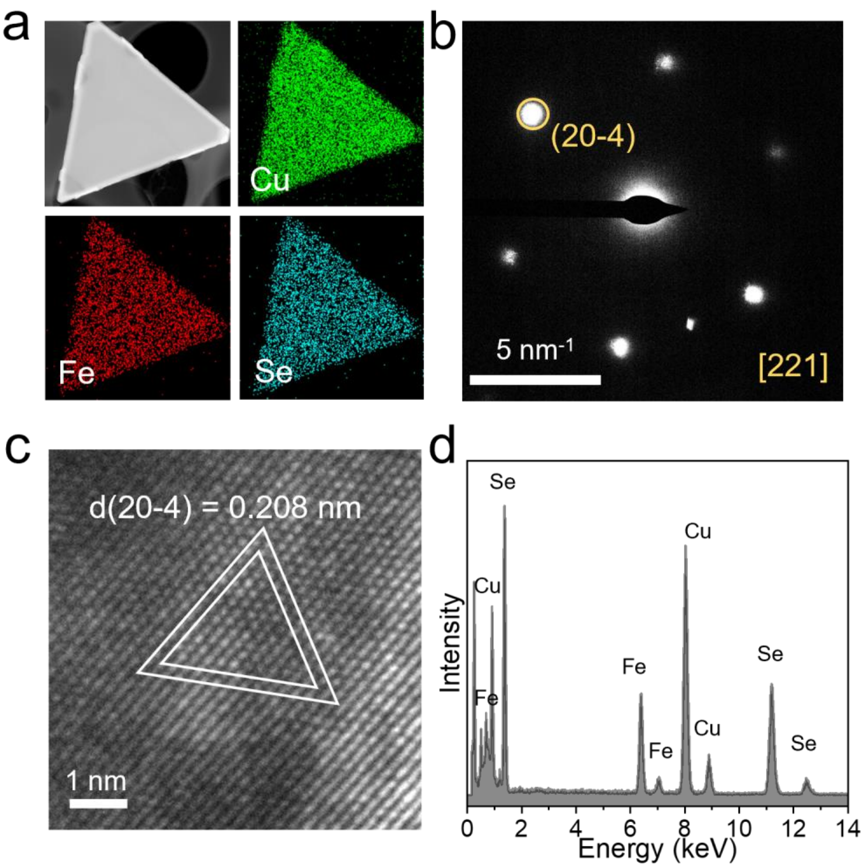

Supplementary Figure 26. Structure characterization of $CuFeSe_2$. a Low magnification TEM image and corresponding EDS elemental mapping of $CuFeSe_2$. **b** SAED pattern of as-synthesized 2D $CuFeSe_2$ nanosheet. **c** Atom-resolution experimental TEM image of $CuFeSe_2$. **d** The EDS spectra of the flake.

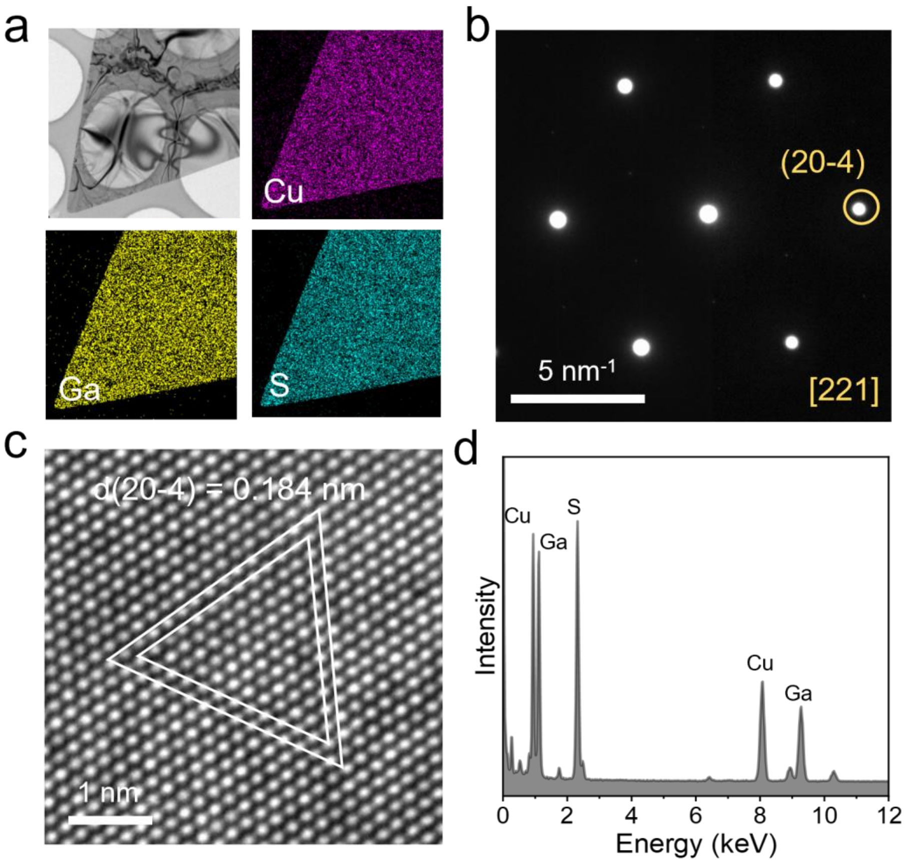

Supplementary Figure 27. Structure characterization of $CuGaS_2$. a Low magnification TEM image and corresponding EDS elemental mapping of $CuGaS_2$. **b** SAED pattern of as-synthesized 2D $CuGaS_2$ nanosheet. **c** Atom-resolution experimental TEM image of $CuGaS_2$. **d** The EDS spectra of the flake.

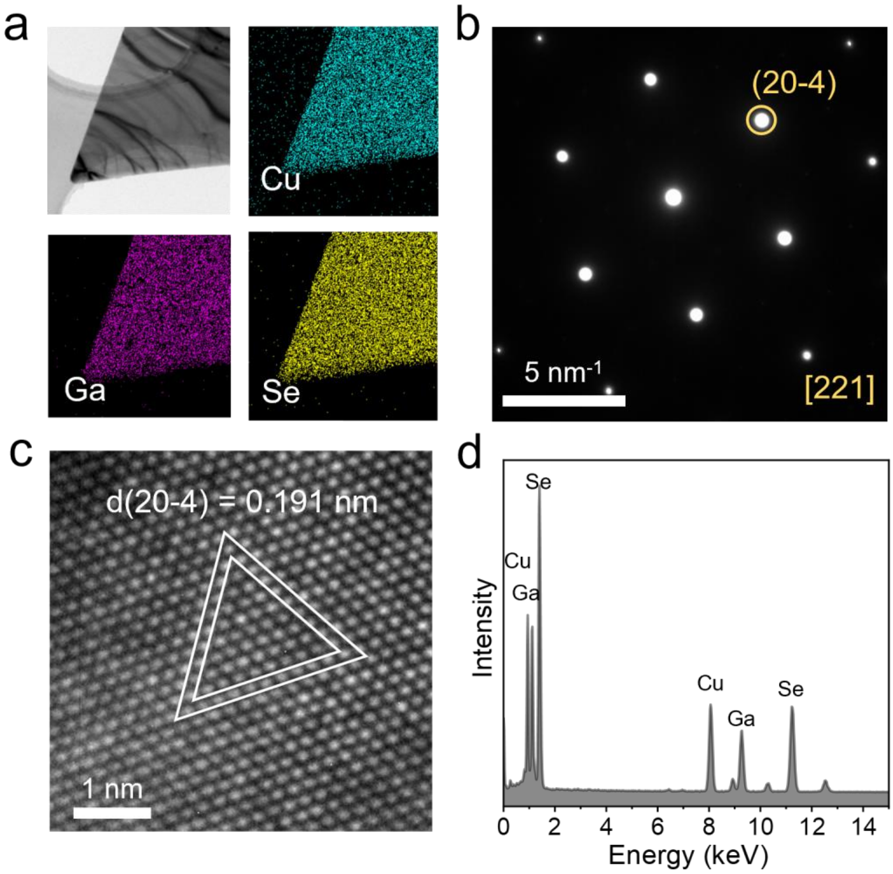

Supplementary Figure 28. Structure characterization of $CuGaSe_2$. a Low magnification TEM image and corresponding EDS elemental mapping of $CuGaSe_2$. **b** SAEDs pattern of as-synthesized 2D $CuGaSe_2$ nanosheet. **c** Atom-resolution experimental TEM image of $CuGaSe_2$. **d** The EDS spectra of the flake.

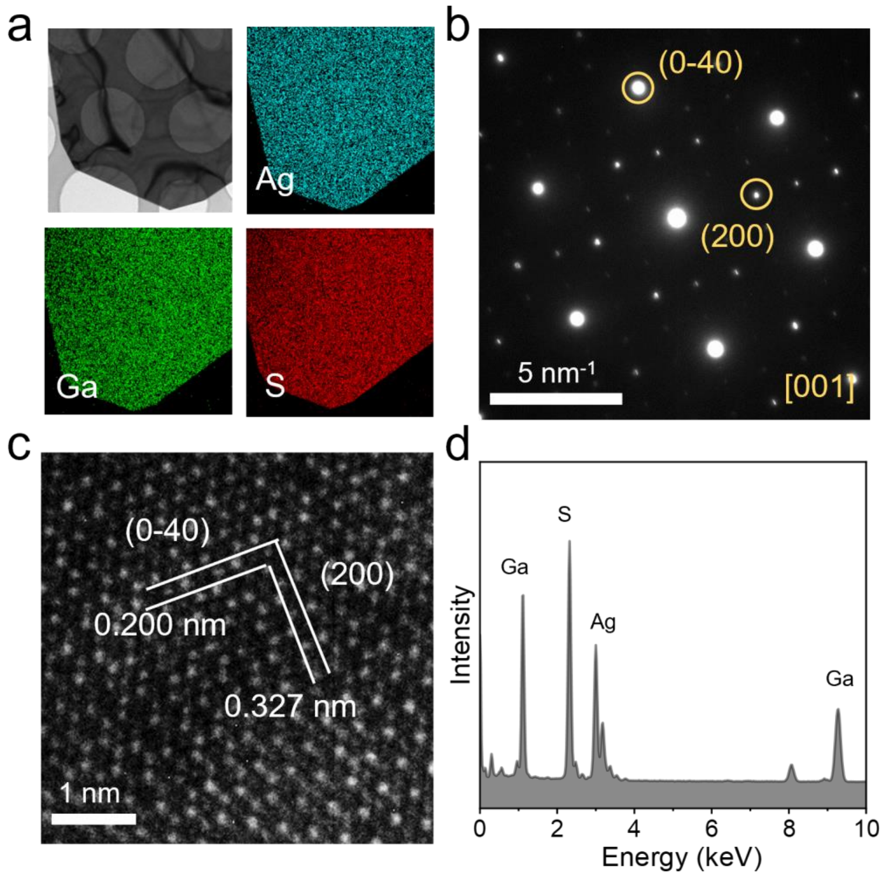

Supplementary Figure 29. Structure characterization of $AgGaS_2$. a Low magnification TEM image and corresponding EDS elemental mapping of $AgGaS_2$. **b** SAED pattern of as-synthesized 2D $AgGaS_2$ nanosheet. **c** Atom-resolution experimental TEM image of $AgGaS_2$. **d** The EDS spectra of the flake.

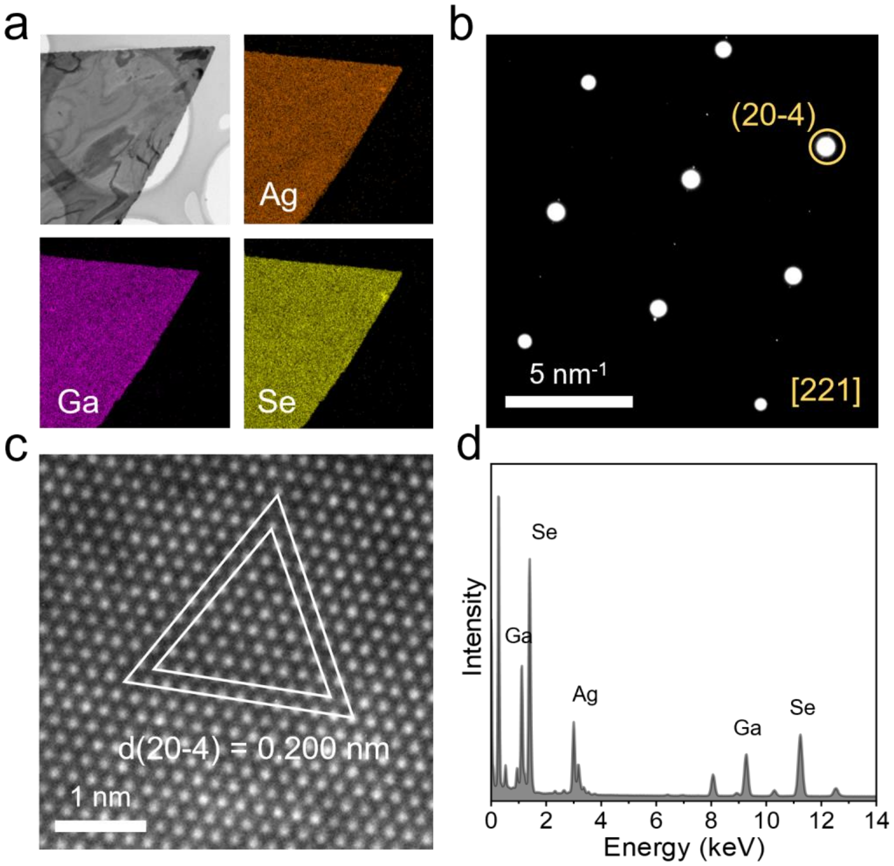

Supplementary Figure 30. Structure characterization of $AgGaSe_2$. a Low magnification TEM image and corresponding EDS elemental mapping of $AgGaSe_2$. **b** SAED pattern of as-synthesized 2D $AgGaSe_2$ nanosheet. **c** Atom-resolution experimental TEM image of $AgGaSe_2$. **d** The EDS spectra of the flake.

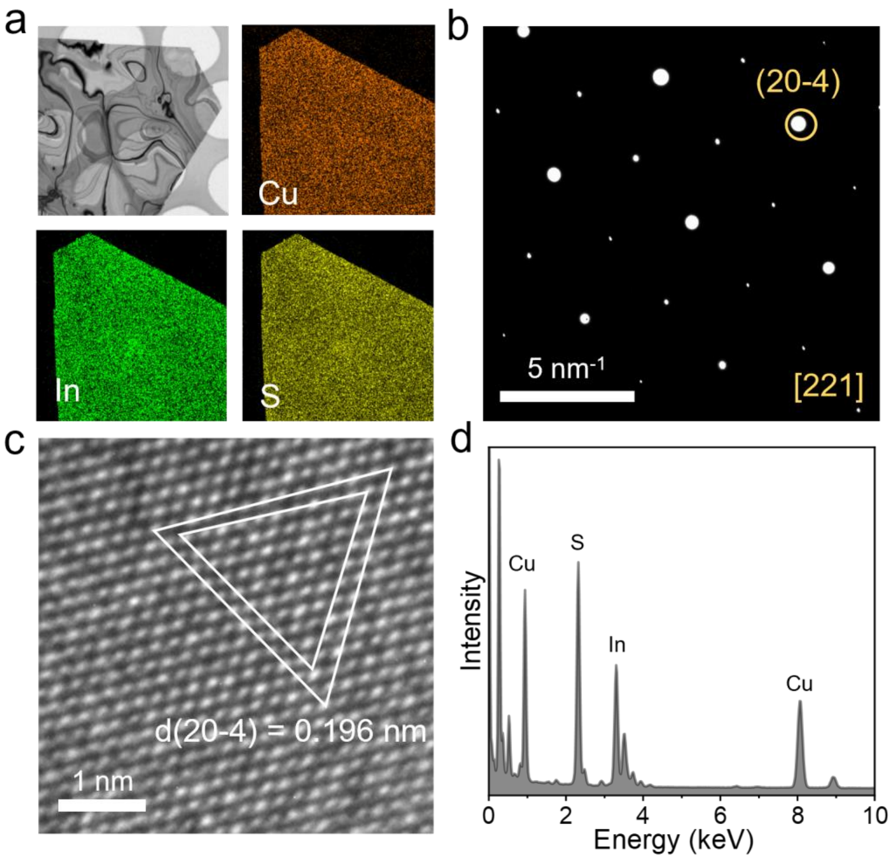

Supplementary Figure 31. Structure characterization of $CuInS_2$. **a** Low magnification TEM image and corresponding EDS elemental mapping of $CuInS_2$. **b** SAED pattern of as-synthesized 2D $CuInS_2$ nanosheet. **c** Atom-resolution experimental TEM image of $CuInS_2$. **d** The EDS spectra of the flake.

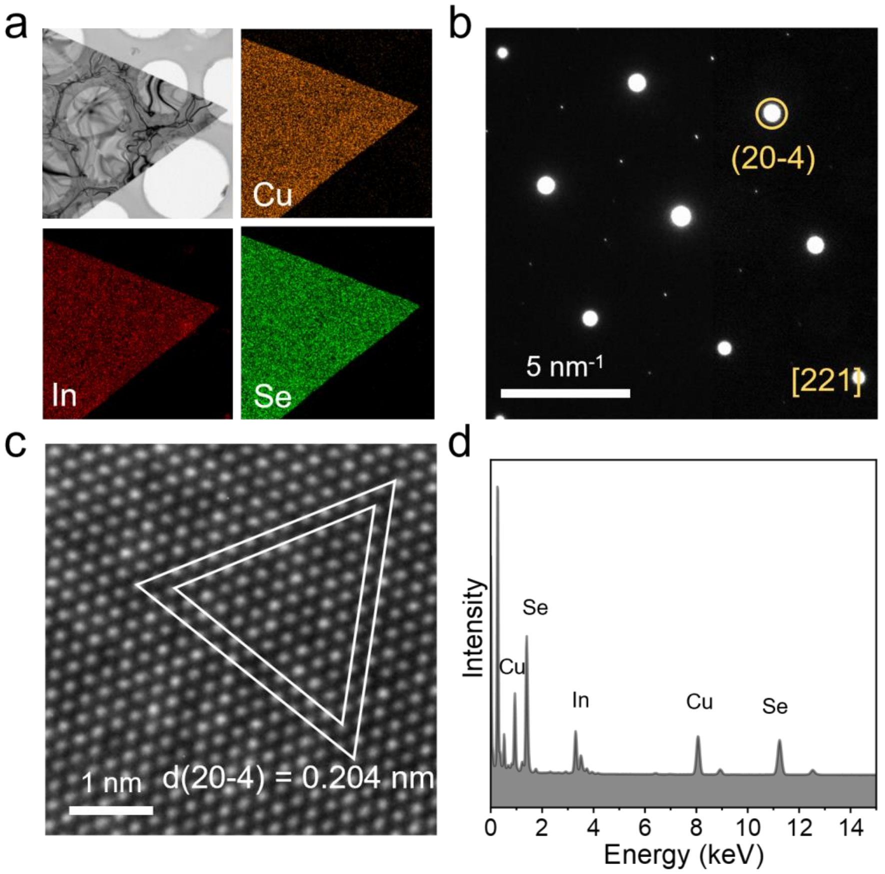

Supplementary Figure 32. Structure characterization of $CuInSe_2$. a Low magnification TEM image and corresponding EDS elemental mapping of $CuInSe_2$. **b** SAED pattern of as-synthesized 2D $CuInSe_2$ nanosheet. **c** Atom-resolution experimental TEM image of $CuInSe_2$. **d** The EDS spectra of the flake.

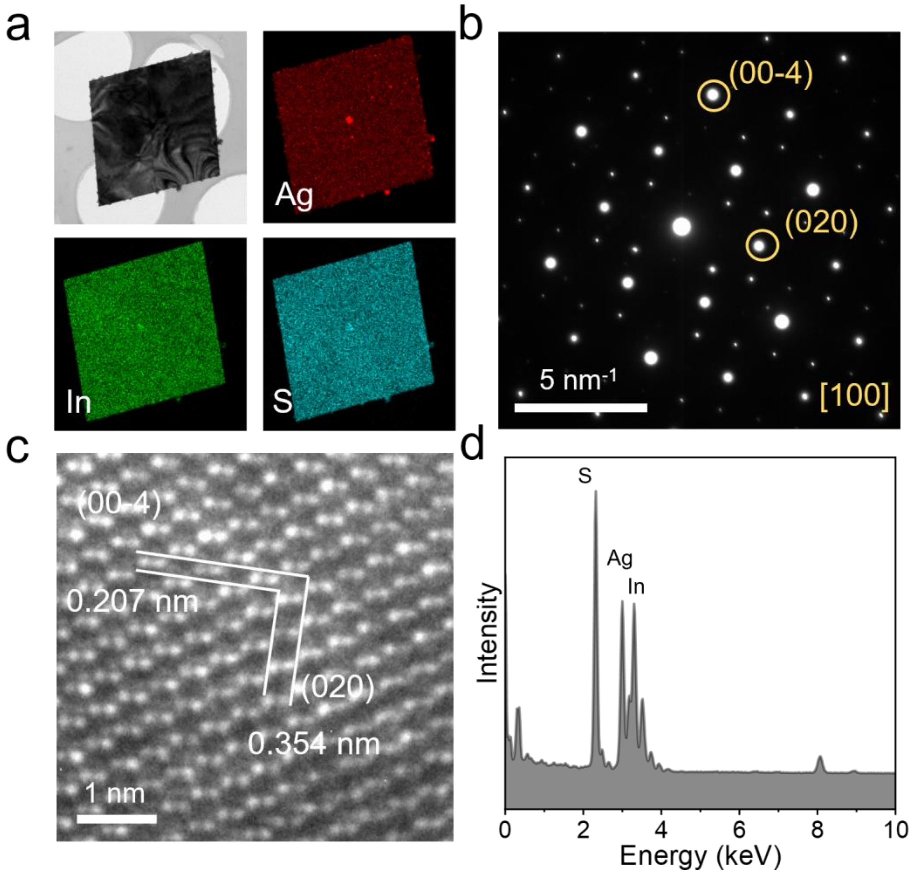

Supplementary Figure 33. Structure characterization of $AgInS_2$. **a** Low magnification TEM image and corresponding EDS elemental mapping of $AgInS_2$. **b** SAED pattern of as-synthesized 2D $AgInS_2$ nanosheet. **c** Atom-resolution experimental TEM image of $AgInS_2$. **d** The EDS spectra of the flake.

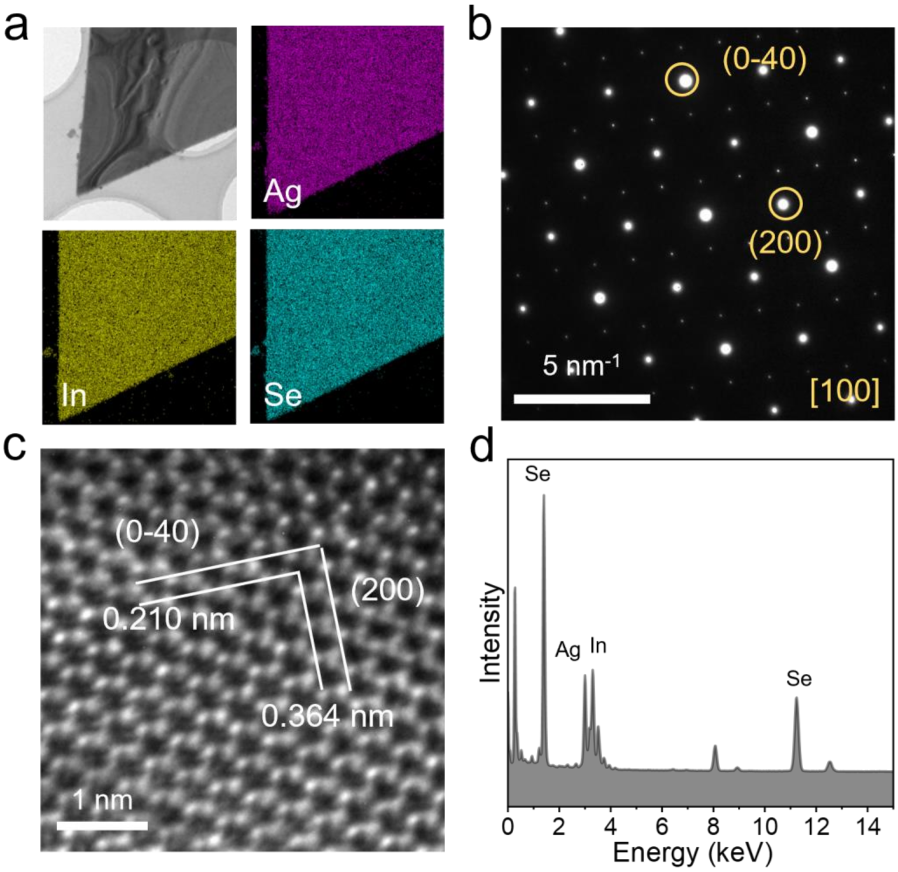

Supplementary Figure 34. Structure characterization of $AgInSe_2$. a Low magnification TEM image and corresponding EDS elemental mapping of $AgInSe_2$. **b** SAED pattern of as-synthesized 2D $AgInSe_2$ nanosheet. **c** Atom-resolution experimental TEM image of $AgInSe_2$. **d** The EDS spectra of the flake.

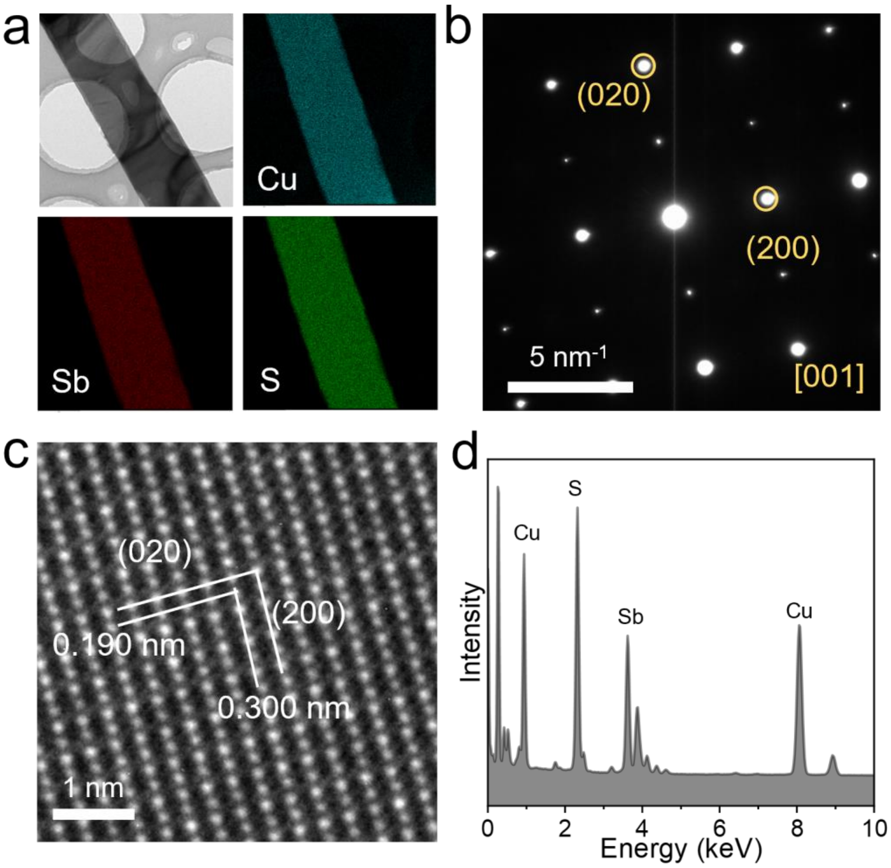

Supplementary Figure 35. Structure characterization of $CuSbS_2$. a Low magnification TEM image and corresponding EDS elemental mapping of $CuSbS_2$. **b** SAED pattern of as-synthesized 2D $CuSbS_2$ nanosheet. **c** Atom-resolution experimental TEM image of $CuSbS_2$. **d** The EDS spectra of the flake.

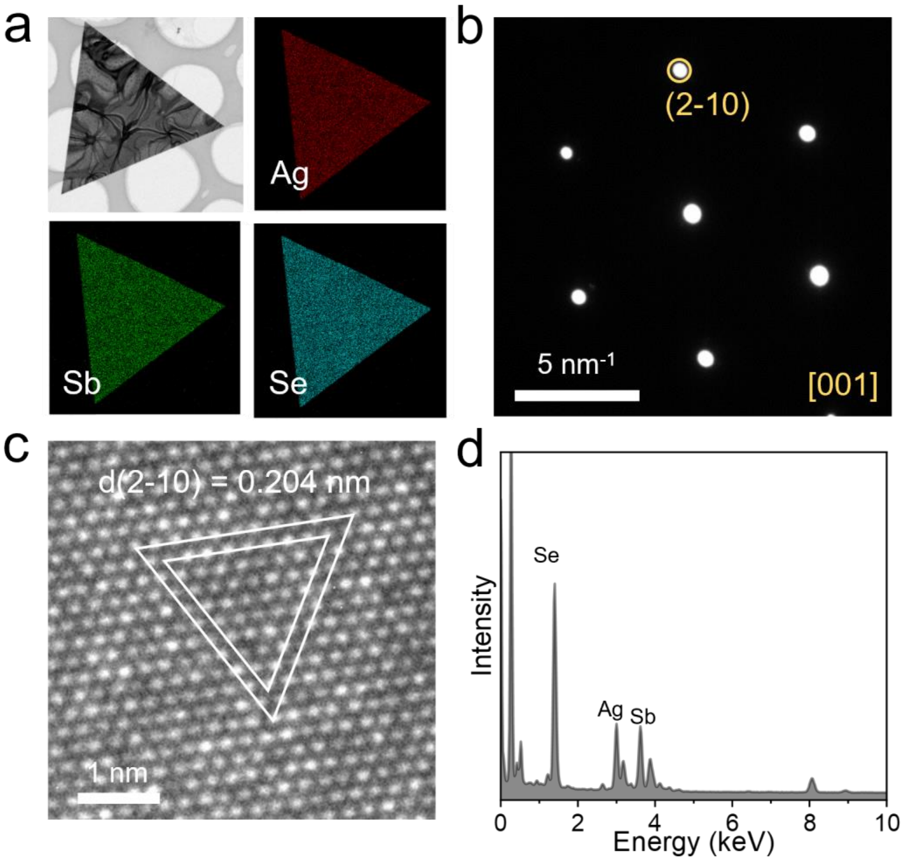

Supplementary Figure 36. Structure characterization of $AgSbSe_2$. a Low magnification TEM image and corresponding EDS elemental mapping of $AgSbSe_2$. **b** SAED pattern of as-synthesized 2D $AgSbSe_2$ nanosheet. **c** Atom-resolution experimental TEM image of $AgSbSe_2$. **d** The EDS spectra of the flake.

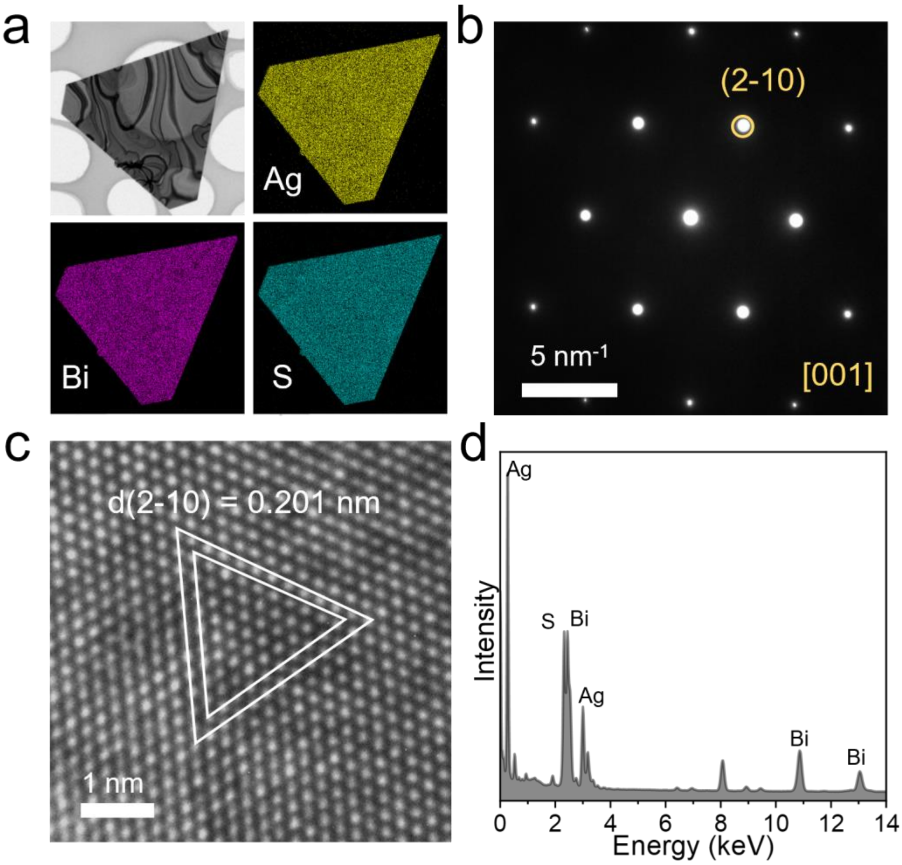

Supplementary Figure 37. Structure characterization of $AgBiS_2$. a Low magnification TEM image and corresponding EDS elemental mapping of $AgBiS_2$. **b** SAED pattern of as-synthesized 2D $AgBiS_2$ nanosheet. **c** Atom-resolution experimental TEM image of $AgBiS_2$. **d** The EDS spectra of the flake.

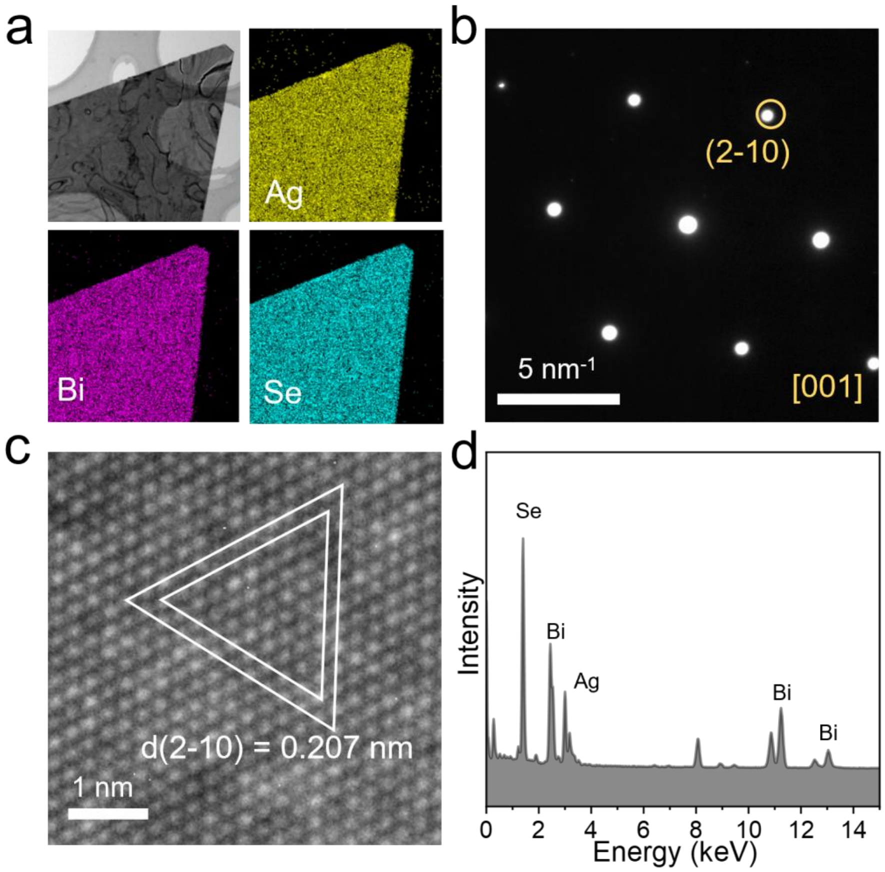

Supplementary Figure 38. Structure characterization of $AgBiSe_2$. a Low magnification TEM image and corresponding EDS elemental mapping of $AgBiSe_2$. **b** SAED pattern of as-synthesized 2D $AgBiSe_2$ nanosheet. **c** Atom-resolution experimental TEM image of $AgBiSe_2$. **d** The EDS spectra of the flake.

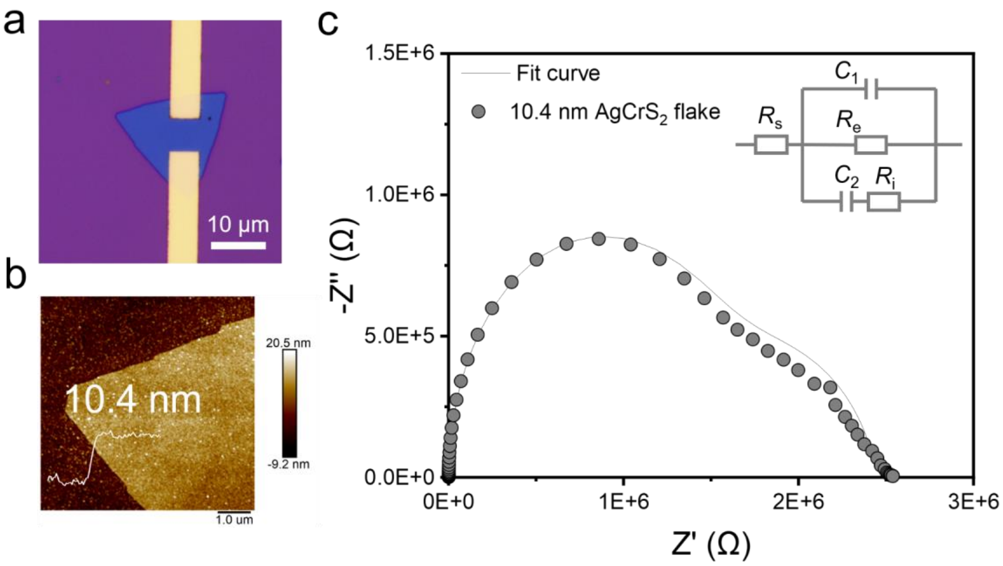

Supplementary Figure 39. Ionic conductivity of the as-grown 2D $AgCrS_2$. a Optical image of the $AgCrS_2$ device with a channel width of 6.09 μm and a channel length of 6.35 μm. **b** AFM image of $AgCrS_2$ nanosheet exhibiting a thickness of 10.4 nm. **c** The Nyquist plot of the device.

Based on the mixed ionic-electronic conductor model, the decoupled ionic conductivity can be obtained by fitting the electrochemical impedance curves. As an example, the Nyquist plot of 10.4 nm $AgCrS_2$ nanosheet can be fitted with two semicircles, and the low-frequency impedance intercept represents electronic impedance (R_e), while the high-frequency intercept corresponds to ionic impedance (R_i). The fitted R_i of the 10.4 nm $AgCrS_2$ is ~5.2 MΩ. The thickness (t) of the flake is 10.4 nm, and the channel width (d) and length (L) of the device are 6.09 μm and 6.35 μm, respectively. Then, the ionic conductivity σ_i at room temperature can be calculated by the following equation:

$$\sigma_i = \frac{L}{R \cdot t \cdot d} = \frac{6.35\ \mu m}{5.2\ M\Omega \cdot 10.4\ nm \cdot 6.09\ \mu m \times 10^{-4}} = 192.8\ mS/cm$$

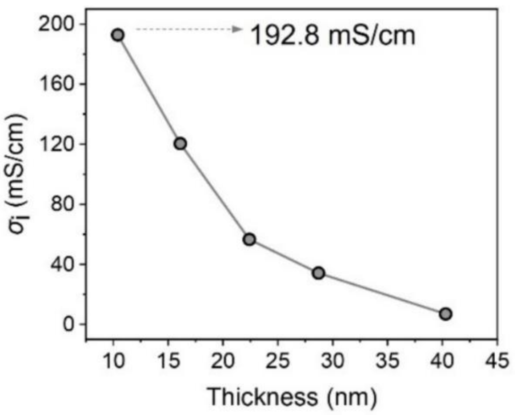

Supplementary Figure 40. Thickness dependence of the ionic conductivity in $AgCrS_2$ nanosheets.

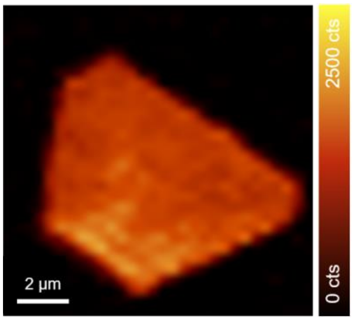

Supplementary Figure 41. The SHG mapping of a $CuScS_2$ nanosheet. SHG signal is sensitive to the local crystal structure, it can be used to reveal the crystals' crystallinity, defects, domain wall, etc[7]. The uniform distribution of the SHG signal demonstrates the good crystallinity and the single-domain characteristics of the as-synthesized $CuScS_2$.

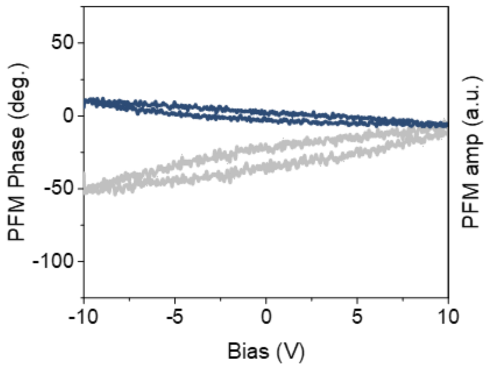

Supplementary Figure 42. The in-plane PFM phase and amplitude hysteresis loop of a $CuScS_2$ nanosheet.

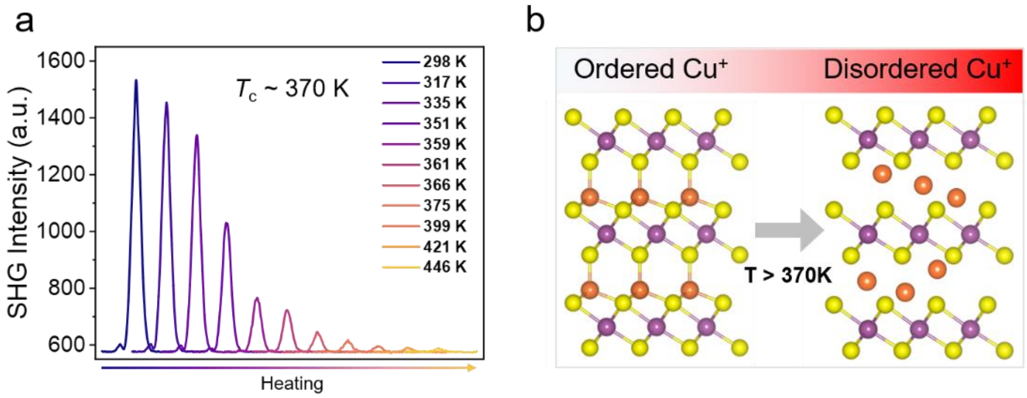

Supplementary Figure 43. Temperature-dependent SHG measurement of a $CuScS_2$ nanosheet. **a** The spectra of SHG signal at different temperatures. **b** Schematic diagram of the arrangement of Cu^+ ion in $CuScS_2$ structure when the temperature is below and beyond the T_C.

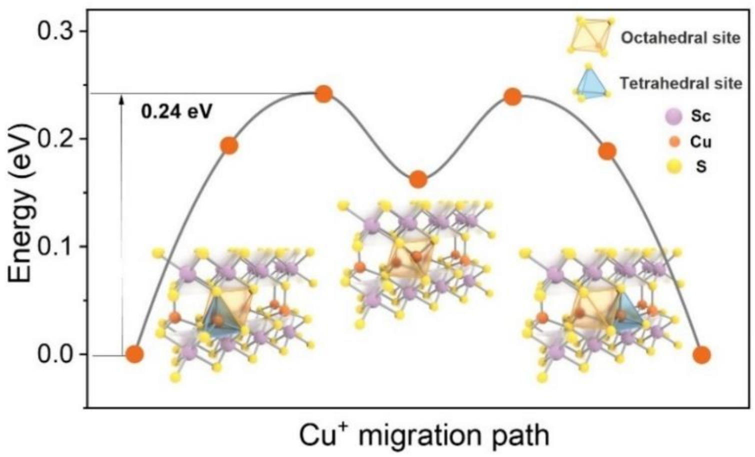

Supplementary Figure 44. The calculated energy barrier for Cu^+ migration in $CuScS_2$.

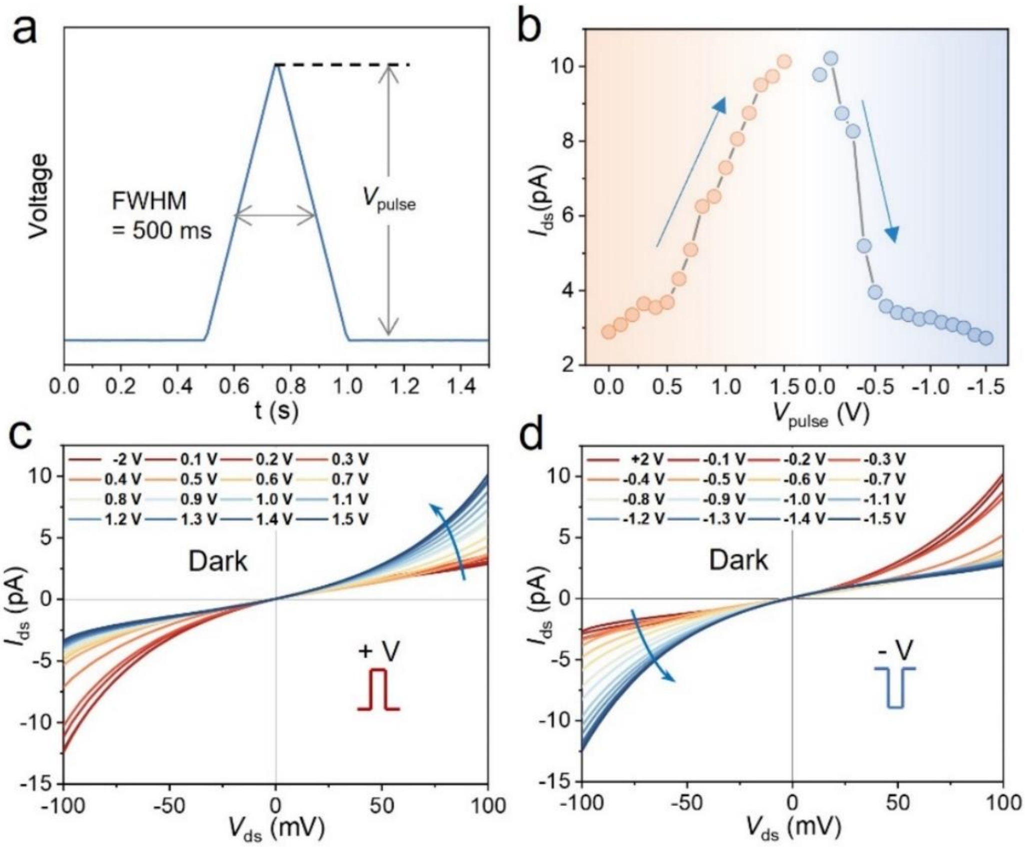

Supplementary Figure 45. Memristor properties in the dark state of $CuScS_2$ device. a Triangular electric pulse. **b** The current variation of the device at 100 mV during positive and negative pulse modulation processes. **c-d** I_{ds}-V_{ds} output characteristics of the $CuScS_2$ device in various positive and negative voltage pulses respectively.

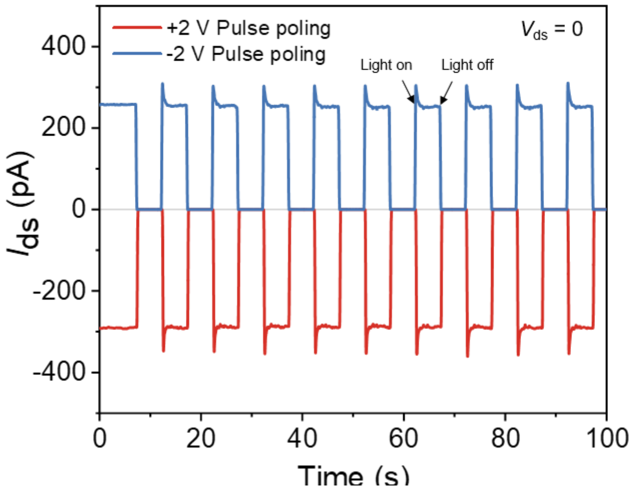

Supplementary Figure 46. The stability of the $CuScS_2$ photovoltaic response. (2 V/0.5 s poling pulse; illumination λ=532 nm, 256.6 mW/cm^2)

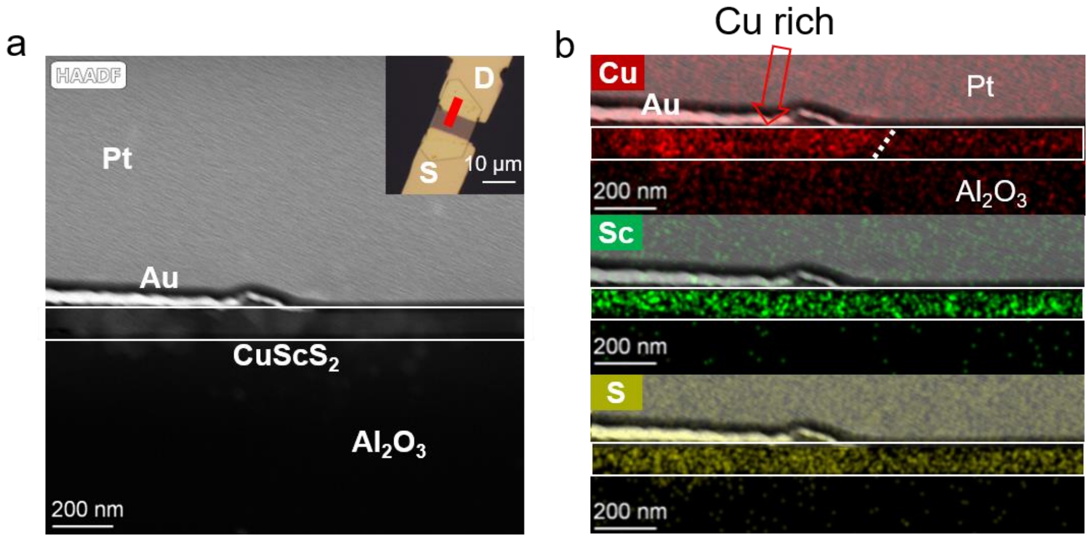

Supplementary Figure 47. Cross-sectional EDS mapping of $CuScS_2$ device after polarization. a SEM image of the $CuScS_2$ device. **b** The EDS mappings of the device after polarization.

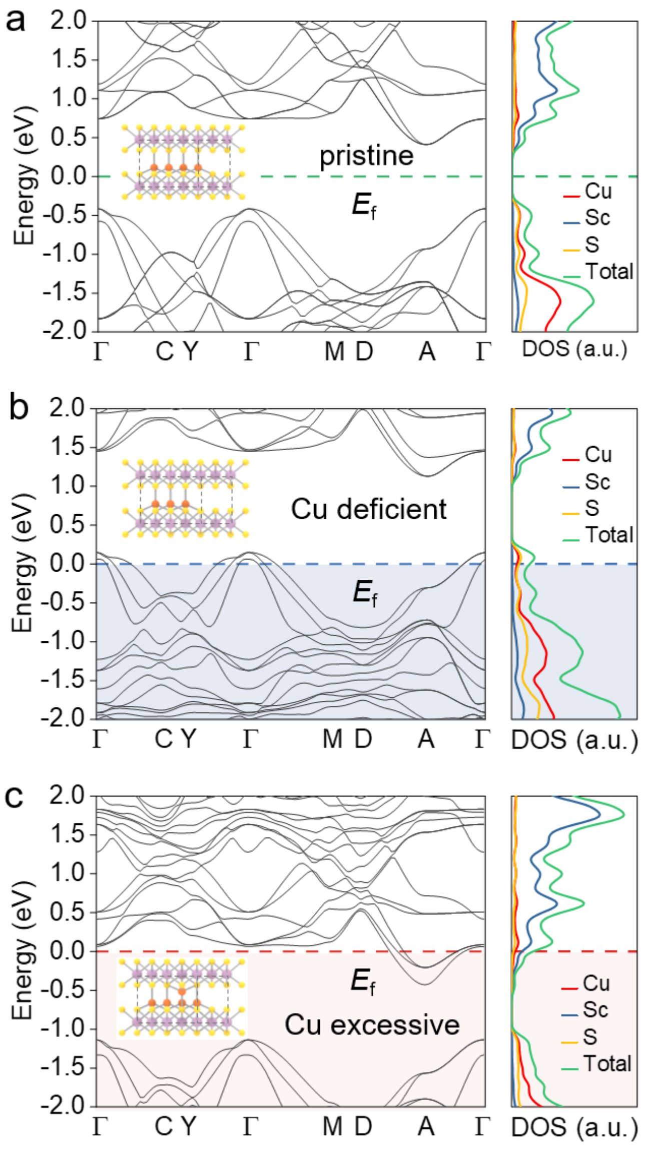

Supplementary Figure 48. Band structures of $CuScS_2$ with different Cu contents. a-c The calculated band structures of the $CuScS_2$ when it is in the intrinsic state (**a**) or when the Cu is deficient (**b**) and excessive (**c**).

Supplementary Tables

Supplementary Table 1. Synthesis conditions of the 20 kinds of 2D AMX_2.

M Element	*AMX_2*	*A Metal Precursor*	*M Metal Precursor*	*Growth Temperature (℃)*	*Carrier Gas*	*Growth Time (min)*
Sc	$CuScS_2$	20 mg CuI	5 mg Sc_2S_3 and 1 mg NaCl	800~850 (50℃/min)	Ar (100 sccm)	5
	$AgScSe_2$	20 mg AgI	5 mg Sc_2Se_3 and 1 mg NaCl	800~850 (50℃/min)	Ar/H_2 (100/5 sccm)	5
Cr	$CuCrS_2$	100 mg CuI	5 mg Cr and 10 mg NaCl	780~900 (50℃/min)	Ar (100 sccm)	5
	$CuCrSe_2$	100 mg CuI	5 mg Cr and10 mg KI	800~850 (50℃/min)	Ar/H_2 (100/5 sccm)	5
	$AgCrS_2$	150 mg AgI	5 mg Cr and 1 mg NaCl	800-850 (50℃/min)	Ar (100 sccm)	5
	$AgCrSe_2$	150 mg AgI	5 mg Cr and 1 mg KI	800~850 (50℃/min)	Ar/H_2 (100/5 sccm)	5
Fe	$CuFeS_2$	70 mg CuI	10 mg Fe and 3 mg KI	700~750 (50℃/min)	Ar (100 sccm)	5
	$CuFeSe_2$	50 mg CuI	10 mg Fe_2O_3 and 3 mg KI	700~750 (50℃/min)	Ar/H_2 (100/5 sccm)	5
Ga	$CuGaS_2$	3 mg Cu_2O and 3 mg NaCl	30 mg Ga	700~750 (50℃/min)	Ar (100 sccm)	10
	$CuGaSe_2$	10 mg Cu_2O and 3 mg KI	30 mg Ga	700~750 (50℃/min)	Ar/H_2 (100/5 sccm)	5
	$AgGaS_2$	5 mg AgI	50 mg Ga	700~750 (50℃/min)	Ar (100 sccm)	5
	$AgGaSe_2$	50 mg AgI	100~150 mg Ga	650~750 (50℃/min)	Ar/H_2 (100/5 sccm)	5
In	$CuInS_2$	3 mg CuO and 1 mg NaCl	50 mg In	700~750 (50℃/min)	Ar (100 sccm)	5
	$CuInSe_2$	10 mg CuO and 5 mg KI	50 mg In and 50 mg In_2O_3	550~600 (50℃/min)	Ar/H_2 (100/5 sccm)	5
	$AgInS_2$	5 mg AgI	50 mg In	700~750 (50℃/min)	Ar (100 sccm)	5
	$AgInSe_2$	5 mg AgI	50 mg In and 50 mg In_2O_3	700~750 (50℃/min)	Ar (100 sccm)	5
Sb	$CuSbS_2$	50 mg CuI	50 mg Sb	540~560 (30℃/min)	Ar/H_2 (50/5 sccm)	/
	$AgSbSe_2$	50 mg AgI	50 mg Sb	540~560 (30℃/min)	Ar/H_2 (50/5 sccm)	/
Bi	$AgBiS_2$	10 mg AgI	50 mg Bi	540~560 (30℃/min)	Ar (100 sccm)	/

	$AgBiSe_2$	10 mg AgI	100 mg Bi	540~560 (30°C/min)	Ar/H_2 (100/5 sccm)	/

Supplementary Table 2. Classification of the as-grown 2D AMX_2 that was neither synthesized by CVD nor obtained by ME in previous.

$CuScS_2$, $AgScSe_2$, $AgCrSe_2$, $CuCrSe_2$, $CuFeS_2$, $CuFeSe_2$, $CuGaS_2$, $CuGaSe_2$, $AgGaS_2$, $AgGaSe_2$, $CuInS_2$, $CuInSe_2$, $AgInS_2$, $AgInSe_2$, $CuSbS_2$, $AgSbSe_2$, $AgBiS_2$, $AgBiSe_2$

(18 kinds)

Supplementary Table 3. Classification of the as-grown 2D AMX_2 according to their possible properties.

Possible magnetic[1–3]	Possible ferroelectric[1,4]	Possible superionic conductor[5,6]
$AgCrS_2$, $AgCrSe_2$, $CuCrS_2$ $CuCrSe_2$, $CuFeS_2$, $CuFeSe_2$	$AgCrS_2$, $AgCrSe_2$, $CuCrS_2$, $CuCrSe_2$, $CuScS_2$	$AgCrS_2$, $AgCrSe_2$, $CuCrS_2$, $CuCrSe_2$, $CuScS_2$, $AgScSe_2$

Supplementary Table 4. The atomic structures of the as-grown 20 kinds of AMX_2.

Materials	Space group	Symmetry	Atomic structures
$AgCrS_2$<br>$AgCrSe_2$<br>$CuCrS_2$<br>$CuCrSe_2$	*R*3*m*	*Non-centrosymmetric*	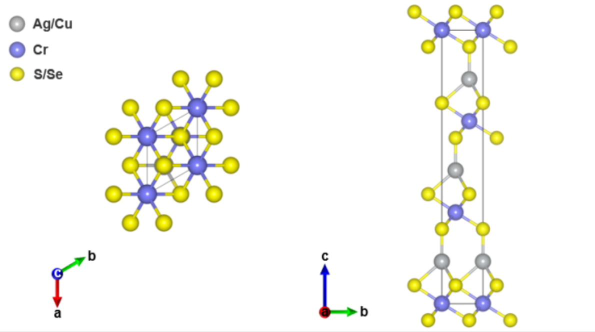
$AgSbSe_2$<br>$AgBiS_2$<br>$AgBiSe_2$	$R\bar{3}m$	Centrosymmetric	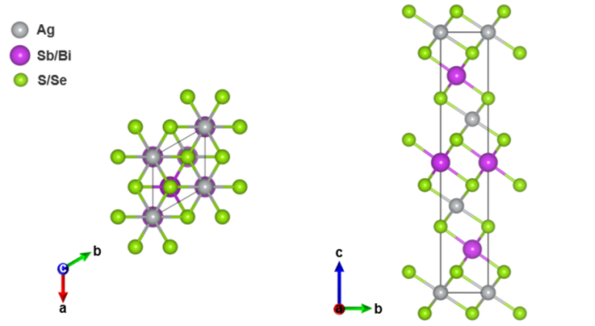

$CuScS_2$	*P3m1*	*Non-centrosymmetric*	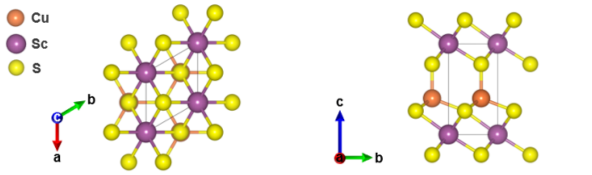
$AgScSe_2$	$P\bar{3}m1$	*Centrosymmetric*	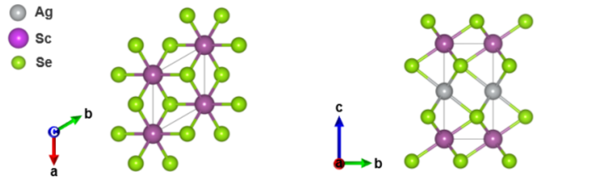
$CuGaS_2$<br>$CuGaSe_2$<br>$AgGaS_2$<br>$AgGaSe_2$<br>$CuInS_2$<br>$CuInSe_2$<br>$CuFeS_2$	$I\bar{4}2d$	*Non-centrosymmetric*	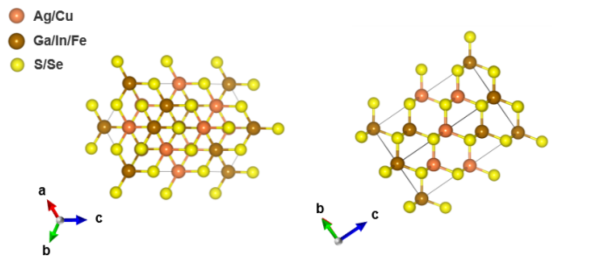
$AgInS_2$<br>$AgInSe_2$	$Pna2_1$	*Non-centrosymmetric*	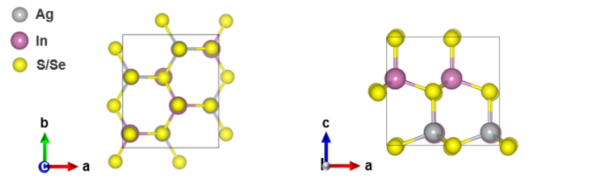
$CuFeSe_2$	$P\bar{4}2c$	*Non-centrosymmetric*	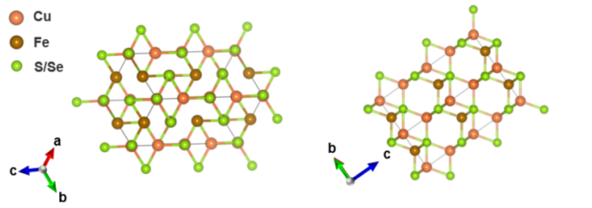
$CuSbS_2$	*Pnma*	*Centrosymmetric*	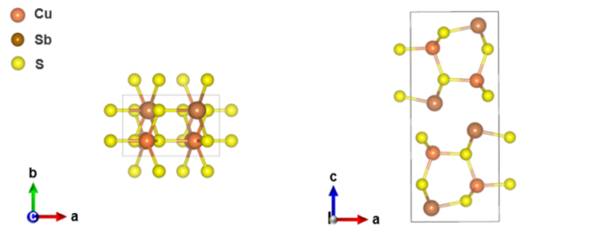

Supplementary Methods

Detailed synthesis descriptions of 2D AMX_2:

$CuScS_2$:

A porcelain boat containing resolidified sulfur precursor was placed upstream and heated to 150℃. 20 mg CuI powder was laid at the bottom of the small quartz tube with a length of 3.5 cm while 5 mg Sc_2S_3 and 1 mg NaCl powder were ground uniformly and placed 1 cm away from the CuI. The mica substrate was placed near the Sc precursors and the one-side sealed small quartz tube was placed downstream with the open side facing upstream. This small tube's bottom is 14 cm away from the heating center of the furnace. During the growth process, a constant flow of 100 sccm Ar was used as the carrier gas. The furnace was heated from room temperature to 800~850 ℃ and maintained for 5 min. Once the growth process finished, the furnace was pushed away for rapid cooling to room temperature. The Sc_2S_3 powder used here was synthesized by vacuum heating the Sc and S powders at 1000℃ for 8 hours.

$AgScSe_2$:

The growth process of $AgScSe_2$ is similar to that of $CuScS_2$. The difference is that the Se precursor was placed upstream, and Sc_2Se_3 and AgI powders were chosen as the metal precursors; 100 sccm Ar and 5 sccm H_2 were used. The Sc_2Se_3 powder used here was obtained by vacuum heating the Sc and Se powders at 1000℃ for 8 hours.

$CuCrS_2$:

The growth process of $CuCrS_2$ is similar to that of $CuScS_2$. The difference is that 100 mg CuI powder was used; 5 mg Cr and 10 mg NaCl powder were ground uniformly and placed 2 cm away from the open side of the small quartz tube; The furnace was heated from room temperature to 780~900 ℃ and maintained for 5 min.

$CuCrSe_2$:

The growth process of $CuCrSe_2$ is similar to that of $CuScS_2$. The difference is that the Se precursor was placed upstream; 100 mg CuI powder was used; 5 mg Cr and 10 mg KI powder were ground uniformly and placed 2 cm away from the open side of the small quartz tube; 100 sccm Ar and 5 sccm H_2 were used.

$AgCrS_2$:

The growth process of $AgCrS_2$ is similar to that of $CuScS_2$. The difference is that 150 mg AgI

powder was placed at the bottom while 5 mg Cr and 1 mg NaCl powder were ground uniformly and placed 2 cm away from the open side of the small quartz tube.

$AgCrSe_2$:

The growth process of $AgCrSe_2$ is similar to that of $CuScS_2$. The difference is that the Se precursor was placed upstream; 150 mg AgI powder was placed at the bottom while 5 mg Cr and 1 mg KI powder were ground uniformly and placed 2 cm away from the open side of the small quartz tube; 100 sccm Ar and 5 sccm H_2 were used.

$CuFeS_2$:

The growth process of $CuFeS_2$ is similar to that of $CuScS_2$. The difference is that 10 mg Fe and 3 mg KI powder were placed at the bottom while 70 mg CuI powder was sandwiched between two micas near the open side of the small quartz tube; The furnace was heated from room temperature to 700~750 ℃ and maintained for 5 min; Natural cooling was used.

$CuFeSe_2$:

The growth process of $CuFeSe_2$ is similar to that of $CuScS_2$. The difference is that the Se precursor was placed upstream; 50mg CuI was used; 10 mg Fe_2O_3 and 3 mg KI powder were ground uniformly and placed 2 cm away from the open side of the small quartz tube; 100 sccm Ar and 5 sccm H_2 were used; The furnace was heated from room temperature to 700~750 ℃ and maintained for 5 min.

$CuGaS_2$:

The growth process of $CuGaS_2$ is similar to that of $CuScS_2$. The difference is that 30 mg Ga was placed at the bottom while 3 mg Cu_2O and 3 mg NaCl powders were ground uniformly and placed 2 cm away from the open side of the small quartz tube; The furnace was heated from room temperature to 700~750 ℃ and maintained for 10 min.

$CuGaSe_2$:

The growth process of $CuGaSe_2$ is similar to that of $CuScS_2$. The difference is that the Se precursor was placed upstream; 30 mg Ga was placed 4.5 cm away from the bottom while 10 mg Cu_2O and 3 mg KI powder were ground uniformly and placed 2 cm away from the open side of the small quartz tube; 100 sccm Ar and 5 sccm H_2 were used; The furnace was heated from room temperature to 700~750 ℃ and maintained for 5 min.

$AgGaS_2$:

The growth process of $AgGaS_2$ is similar to that of $CuScS_2$. The difference is that 50 mg Ga was placed at the bottom while 5 mg AgI powder was placed 2 cm away from the open side of the small quartz tube; The furnace was heated from room temperature to 700~750 ℃ and maintained for 5 min.

$AgGaSe_2$:

The growth process of $AgGaSe_2$ is similar to that of $CuScS_2$. The difference is that the Se precursor was placed upstream; 100~150 mg Ga was placed at the bottom while 50 mg AgI powder was placed 2 cm away from the open side of the small quartz tube; 100 sccm Ar and 5 sccm H_2 were used; The furnace was heated from room temperature to 650~750 ℃ and maintained for 5 min.

$CuInS_2$:

The growth process of $CuInS_2$ is similar to that of $CuScS_2$. The difference is that 50 mg In powder was placed at the bottom while 3 mg CuO powder and 1mg NaCl powder were ground uniformly and placed 2 cm away from the open side of the small quartz tube; The furnace was heated from room temperature to 700~750 ℃ and maintained for 5 min.

$CuInSe_2$:

The growth process of $CuInSe_2$ is similar to that of $CuScS_2$. The difference is that the Se precursor was placed upstream; Mixed 50 mg In_2O_3 and 50 mg In powders were placed at the bottom while 10 mg CuO and 5 mg KI powder were ground uniformly and placed 2 cm away from the open side of the small quartz tube; 100 sccm Ar and 5 sccm H_2 were used; The furnace was heated from room temperature to 550~600 ℃ and maintained for 5 min.

$AgInS_2$:

The growth process of $AgInS_2$ is similar to that of $CuScS_2$. The difference is that 50 mg In powder was placed at the bottom while 5 mg AgI powder was placed 2 cm away from the open side of the small quartz tube; The furnace was heated from room temperature to 700~750 ℃ and maintained for 5 min.

$AgInSe_2$:

The growth process of $AgInSe_2$ is similar to that of $CuScS_2$. The difference is that the Se precursor was placed upstream of the quartz tube; Mixed 50 mg In_2O_3 and 50 mg In powder were placed at the bottom while 5 mg AgI powder was placed 2 cm away from the open side of the small quartz tube; The furnace was heated from room temperature to 700~750 ℃ and maintained for 5 min.

$CuSbS_2$:

The growth process of $CuSbS_2$ is similar to that of $CuScS_2$. The difference is that 50 mg Sb powder was placed at the bottom of the small quartz tube and 50 mg CuI powder was placed above Sb powder separated by a piece of sapphire. Three mica substrates were placed horizontally near the open side of the small quartz tube, while the small tube was placed downstream with the open side facing downstream. This small tube's open side is 14 cm away from the heating center of the furnace. 50 sccm Ar and 5 sccm H_2 were used. The growth temperature was 540~560 ℃ with a ramp rate of 30 ℃/min. Once reaching the desired growth temperature, the process was terminated and the furnace was cooled naturally to room temperature.

$AgSbSe_2$:

The growth process of $AgSbSe_2$ is similar to that of $CuSbS_2$. The difference is that the Se precursor was placed upstream; 50 mg AgI powder was placed near Sb powder.

$AgBiS_2$:

The growth process of $AgBiS_2$ is similar to that of $CuSbS_2$. The difference is that 50 mg Bi powder was placed at the bottom while 10 mg AgI powder was placed near Bi powder; 100 sccm Ar was used.

$AgBiSe_2$:

The growth process of $AgBiSe_2$ is similar to that of $CuSbS_2$. The difference is that the Se precursor was placed upstream. 100 mg Bi powder was placed at the bottom while 10 mg AgI powder was placed near the Bi powder; 100 sccm Ar and 5 sccm H_2 were used.

Supplementary References

1. Zhong, T., Li, X., Wu, M. & Liu, J.-M. Room-temperature multiferroicity and diversified magnetoelectric couplings in 2D materials. *Natl. Sci. Rev.* **7**, 373–380 (2020).
2. Kanematsu, T., Okamoto, Y. & Takenaka, K. Large magnetic-field-induced strain at the magnetic order transition in triangular antiferromagnet $AgCrS_2$. *Appl. Phys. Lett.* **118**, 142404 (2021).
3. Rasch, J. C. E. *et al.* Magnetoelastic coupling in the triangular lattice antiferromagnet $CuCrS_2$. *Phys. Rev. B* **80**, 7 (2009).
4. Abrahams, S. C. Inorganic structures in space group *P*3*m*1; coordinate analysis and systematic prediction of new ferroelectrics. *Acta Crystallogr. B* **64**, 426–437 (2008).
5. Li, B. *et al.* Liquid-like thermal conduction in intercalated layered crystalline solids. *Nat. Mater.* **17**, 226–230 (2018).
6. Niedziela, J. L. *et al.* Selective breakdown of phonon quasiparticles across superionic transition in $CuCrSe_2$. *Nat. Phys.* **15**, 73–78 (2019).
7. Khan, A. R. *et al.* Optical harmonic generation in 2D materials. *Adv. Funct. Mater.* **32**, 2105259 (2022).
